# Quadrivalent mosaic HexaPro-bearing nanoparticle vaccine protects against infection of SARS-CoV-2 variants

**DOI:** 10.1038/s41467-022-30222-w

**Published:** 2022-05-13

**Authors:** Yin-Feng Kang, Cong Sun, Jing Sun, Chu Xie, Zhen Zhuang, Hui-Qin Xu, Zheng Liu, Yi-Hao Liu, Sui Peng, Run-Yu Yuan, Jin-Cun Zhao, Mu-Sheng Zeng

**Affiliations:** 1 State Key Laboratory of Oncology in South China, Collaborative Innovation Center for Cancer Medicine, Guangdong Key Laboratory of Nasopharyngeal Carcinoma Diagnosis and Therapy, Department of Experimental Research, Sun Yat-sen University Cancer Center (SYSUCC), Sun Yat-sen University, Guangzhou, 510060 P. R. China; 2 State Key Laboratory of Respiratory Disease, National Clinical Research Center for Respiratory Disease, Guangzhou Institute of Respiratory Health, The First Affiliated Hospital of Guangzhou Medical University, Guangzhou, 510182 P. R. China; 3 Cryo-electron Microscopy Center, Southern University of Science and Technology, Shenzhen, 518000 P. R. China; 4 Institute of Precision Medicine, Clinical Trials Unit, The First Affiliated Hospital of Sun Yat-sen University, Guangzhou, 510080 P. R. China; 5 Department of Endocrinology, The First Affiliated Hospital of Sun Yat-sen University, Guangzhou, 510080 P. R. China; 6 Guangdong Provincial Institution of Public Health, Guangdong Provincial Center for Disease Control and Prevention, Guangzhou, 511430 P. R. China; 7 Guangzhou Laboratory, Bio-island, Guangzhou, 510320 P. R. China; 8 Guangdong-Hong Kong Joint Laboratory for RNA Medicine, Guangzhou, 510120 P. R. China

**Keywords:** Protein vaccines, SARS-CoV-2, Viral infection

## Abstract

Emerging SARS-CoV-2 variants of concern (VOCs) harboring multiple mutations in the spike protein raise concerns on effectiveness of current vaccines that rely on the ancestral spike protein. Here, we design a quadrivalent mosaic nanoparticle vaccine displaying spike proteins from the SARS-CoV-2 prototype and 3 different VOCs. The mosaic nanoparticle elicits equivalent or superior neutralizing antibodies against variant strains in mice and non-human primates with only small reduction in neutralization titers against the ancestral strain. Notably, it provides protection against infection with prototype and B.1.351 strains in mice. These results provide a proof of principle for the development of multivalent vaccines against pandemic and potential pre-emergent SARS-CoV-2 variants.

## Abstract (web summary)

Emerging SARS-CoV-2 variants with multiple mutations raise concerns on vaccine effectiveness. Here, Kang *et al*. report that a quadrivalent mosaic nanoparticle vaccine displaying spike proteins from the SARS-CoV-2 prototype and three different VOCs confer protection against SARS-CoV-2 variants in mice.

## Introduction

With the prolonged COVID-19 pandemic, emergent SARS-CoV-2 variants gradually become a major threat for disease control (https://www.who.int/en/activities/tracking-SARS-CoV-2-variants/, accessed 13 April 2022). Currently, the emergence of five VOCs, including B.1.1.7 (Alpha), P.1 (Gamma), B.1.351 (Beta), B.1.617.2 (Delta), and Omicron (B.1.1.529) have drawn broad attention due to their altered pathogenicity and transmissibility as well as reduced sensitivity to vaccine-elicited sera or monoclonal antibodies (mAbs), endangering worldwide efforts to control COVID-19^1–8^. Moreover, rapidly increasing reports of emerging SARS-CoV-2 variants brought additional uncertainty surrounding the efficacy of existing vaccines^9^, which requires the development of new vaccine candidates against circulating VOCs and other variants.

SARS-CoV-2 spike protein plays a critical role in viral attachment and fusion with the host cell^10^. Therefore, the majority of antibodies that neutralize viral infection target the spike protein, making it an ideal antigen to elicit potent protective immunity from SARS-CoV-2 infection. However, mutations from the SARS-CoV-2 variants located on the spike protein extensively affected its antigenicity, leading to broad antibody resistance and immune escape from vaccinate-elicited sera. It has been reported that the D614G substitution in the spike protein of SARS-CoV-2 has become dominant globally, displaying increased infectivity and transmissibility with slightly increased susceptibility to neutralization by sera from convalescent and vaccinated individual and spike-specific mAbs^11–14^. In addition, the B.1.1.7 variant (Alpha) harboring the N501Y mutation shows reduced antibody binding to the spike protein along with increased ACE2 binding^15,16^. An increasing body of evidence indicates that vaccines still remained remarkably effective against the B.1.1.7 variant^9,17^. However, the B.1.351 (Beta), B.1.1.529 (Omicron), and P.1 (Gamma) variants with K417N/T, E484K/A, and N501Y mutations in the receptor-binding domain (RBD) of spike protein, especially the E484K substitution, were confirmed to exhibit markedly reduced neutralization sensitivity to vaccine-elicited sera and various neutralizing antibodies^18,19^. While the emerging B.1.617.2 (Delta) variant with L452R and T478K mutations in the RBD gradually replaced other VOCs to become the dominant variant spreading worldwide, only partial escape from current COVID-19 vaccines was observed in this variant^20,21^. Further studies on the molecular mechanism of the mutation-driving immune escape have identified the role of spike mutations in undermining antibody or receptor affinity^16,22^, suggesting that the humoral immune response elicited by vaccines based on the wild-type virus spike could be vulnerable to emergent and potential new SARS-CoV-2 variants. However, there are few studies on the efficacy of mutant spike protein-based antigens, which could be critical for developing modified SARS-CoV-2 vaccines for primary vaccination to establish viral immunity or booster shots to enhance protection from new variants.

Recombinant multivalent nanoparticle protein vaccines have undergone rapid development in recent years, and are considered a powerful platform for SARS-CoV-2 vaccination because they induce more robust and durable humoral immunity than monomeric antigens^23–26^. We and other groups previously used the SpyTag/SpyCatcher conjugation system to achieve antigen display on the surface of nanoparticle and develop the SARS-CoV-2 nanoparticle vaccine for enhanced immunogenicity^27–31^. Saunders et al.^32^ reported that macaque vaccination with a multimeric SARS-CoV-2 RBD-based nanoparticle vaccine using transpeptidase sortase A covalently links RBD to the H. pylori ferritin nanoparticle induced cross-neutralizing antibody response against pandemic and pre-emergent coronaviruses. Self-assembled nanoparticle platform, like 24-subunit Ferritin, 60-subunit E2p and I3-01, and 120-subunit two-component I53-50 protein nanoparticles have been widely utilized as engineered antigen presentation nanocarrier for the display of SARS-CoV-2 spike ectodomain or RBD antigens by genetic fusion method to increase the humoral immunity in vivo^23–25,33,34^. Moreover, the versatility of this platform in antigen presentation was demonstrated during vaccine development using antigens with variable virus subtypes. By displaying hemagglutinins from diverse influenza strains coherently on the surface, such nanoparticle vaccines could elicit broad protection from a wide range of virus subtypes^35,36^, which could be an ideal scheme for dealing with the pandemic of emergent SARS-CoV-2 variants.

In this study, we designed a quadrivalent mosaic SARS-CoV-2 nanoparticle vaccine comprising spike proteins from the SARS-CoV2 prototype and three major variants, and investigated its potency and breadth of protective humoral immune responses against SARS-CoV-2 variants in detail.

## Results

### Design and characterization of quadrivalent mosaic SARS-CoV-2 HexaPro-based nanoparticle vaccines

**Fig. 1:**
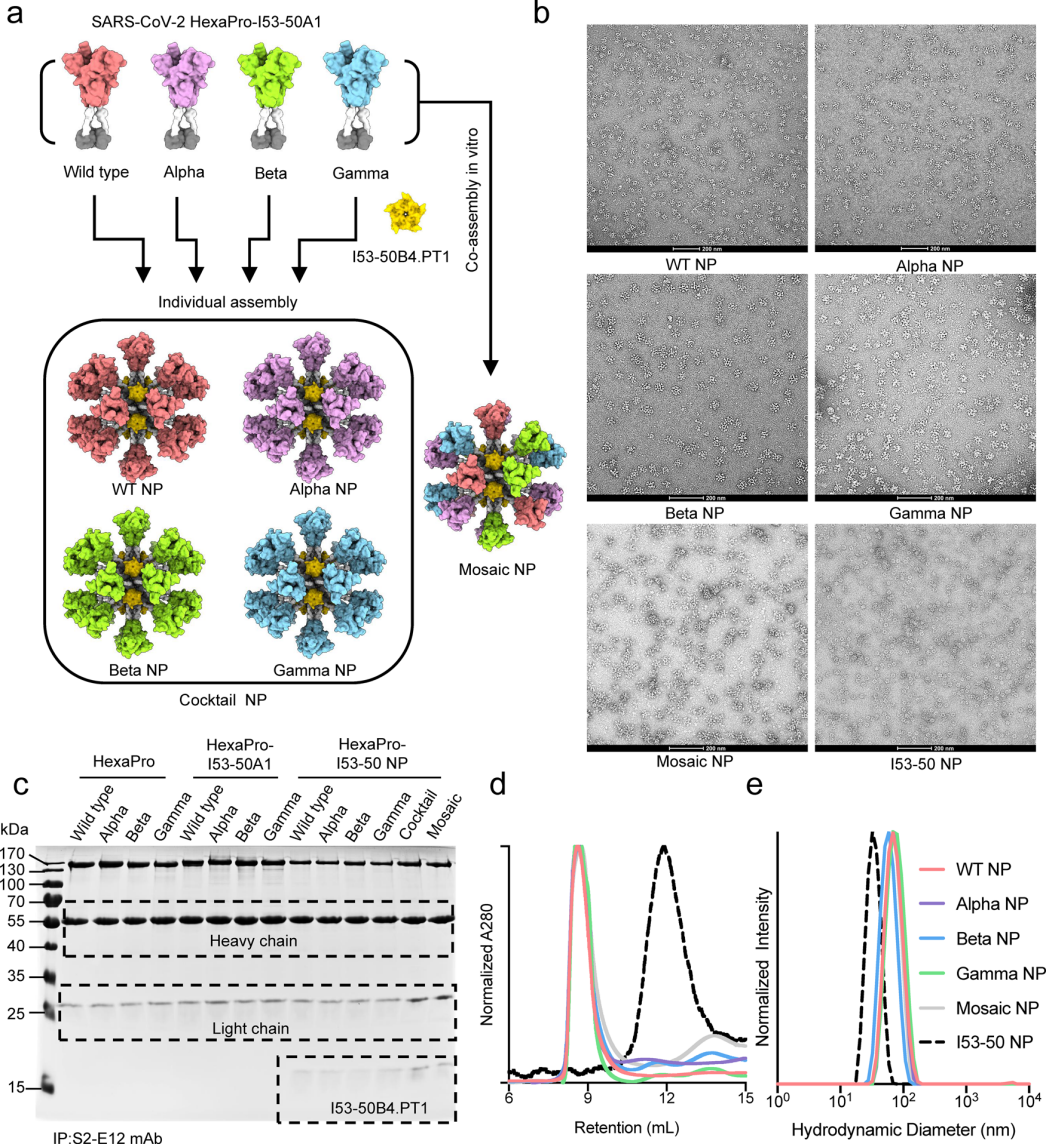
Molecular design and characterization of SARS-CoV-2 HexaPro-based nanoparticle vaccines. **a** Schematic diagram of SARS-COV-2 HexaPro-bearing nanoparticle vaccines. Cocktail NP includes the four individual HexaPro-I53-50 NPs from SARS-CoV-2 prototype, B.1.1.7 (Alpha), P.1 (Gamma), and B.1.351 (Beta) variants. Mosaic NP includes the four HexaPro from SARS-CoV-2 prototype, B.1.1.7, P.1, and B.1.351 variants fused to I53-50A1 and assembled with I53-50B.4PT1 in vitro. The figure was created with PyMOL-2.5.2 and UCSF ChimeraX-1.1.1. **b** Negative-staining electron microscopy images of SARS-CoV-2 HexaPro-based immunogens and individual I53-50 nanoparticles. Scale bar, 200 nm. Similar assay was conducted multiple times to verify the integrity and efficacy of the self-assembled HexaPro-based nanoparticle vaccine. **c** Immunoprecipitation analysis of purified HexaPro immunogens and SARS-CoV-2 RBD-specific S2-E12 neutralizing monoclonal antibody. IP immunoprecipitation. Similar analysis has beenconducted at least three times. **d** Size exclusion chromatography profiles of SARS-CoV-2 HexaPro-based nanoparticles and individual I53-50 nanoparticles on a Superose 6 Increase 10/300 GL column. **e** Dynamic light scattering of SARS-CoV-2 HexaPro-based nanoparticles and individual I53-50 nanoparticles recorded using a Zetasizer Ultra instrument. Source data are provided as a Source Data file.

SARS-CoV-2 emerged and circulates in both humans and animals around the world, including strains classified as variants of concern (VOC), variants of interest (VOI), and variants under monitoring (VUM). For example, P.1 (Gamma), B.1.351 (Beta) and B.1.1.529 (Omicron) variants, showed a reduced sensitivity to SARS-CoV-2 spike-specific mAbs as well as sera from convalescent or vaccinated individuals^9^, representing a serious threat of future outbreaks. Here, we used computational and structure-guided design to develop spike protein nanoparticles by genetically fusing prefusion-stabilized HexaPro of SARS-CoV-2 prototype and 3 VOCs (including Alpha, Beta, and Gamma variants) to the N-terminus of trimeric I53-50A using a flexible and rigid linker (Fig. 1a). Compared to S-2P, another optimized spike construct widely used in mRNA, vector and subunit vaccine design, HexaPro increased the yield and temperature stability of the recombinant protein, while retaining the prefusion conformation, as well as preserving the native antigenicity^37^. After transfection in Expi293F^TM^ suspension cells, the recombinant HexaPro-I53-50A protein was expressed and purified by immobilized metal affinity chromatography and size-exclusion chromatography (SEC). An excess pentameric I53-50B.4PT1 component was added at an equimolar ratio to the four purified HexaPro-I53-50A mixtures for in vitro co-assembly, resulting in quadrivalent mosaic immunogens (Mosaic NP) that co-displayed the 20 trimeric HexaPro of SARS-CoV-2 prototype and 3 VOCs on the surface of icosahedral I53-50 nanoparticles. Similarly, we independently mixed and assembled four HexaPro-based nanoparticle vaccines that displayed the HexaPro individually as mentioned above. In addition, we acquired a cocktail immunogen (Cocktail NP) in which equimolar amounts of four HexaPro-based nanoparticles were individually assembled and mixed together in vitro (Fig. 1a). Negative-stain electron microscopy showed that the purified HexaPro-based nanoparticle exhibited an icosahedral architecture with globular protrusions, which confirmed that HexaPro was presented on the surface of I53-50 nanoparticles (Fig. 1b). To examine the antigenicity of HexaPro-based immunogens, we performed immunoprecipitation analysis using the S2-E12 antibody against HexaPro. The S2-E12 antibody is an ultrapotent RBM antibody with high binding affinity and neutralization potency against broad SARS-related betacoronavirus clades^38,39^. As shown in Fig. 1c, all trimeric HexaPro and HexaPro-I53-50A and HexaPro-I53-50 nanoparticle of SARS-CoV-2 wild type and variants were immunoprecipitated equivalently by S2-E12. We also conducted ELISA analysis to characterize the antigenicity of Mosaic NP using a panel of SARS-CoV-2 wild-type N-terminal domain (NTD) or RBD-directed mAbs. Beta NP and Gamma NP specifically reacted with REGN-10933, Regdanvimab, S2-E12, COVA1-16, CR3022, COV2-2196, and REGN-10987 antibodies. However, these two immunogens didn’t bind to S2-H14, S2-M11, CB6, IgG1-ab1, P2B-2F6, and Fab 2-15 antibodies. Except for S2-H14 and 4A8 antibodies, Alpha NP reacted with the selected mAbs. Mosaic NP bound strongly to all tested mAbs, indicating that Mosaic NP retained desired conformation and antigenicity (Supplementary Fig. 1). SEC and dynamic light scattering profiles demonstrated that the purified individual HexaPro-I53-50 and mosaic HexaPro-I53-50 nanoparticles were homogenous, with larger molecular weight and hydrodynamic diameter than I53-50 NP (Fig. 1d, e). These results suggested that the trimeric I53-50A fusion protein bearing HexaPro and pentameric I53-50B.4PT1 successfully self-assembled into well-ordered icosahedral nanoparticles. To evaluate the antigenic properties of the four purified individual HexaPro-I53-50 nanoparticles of SARS-CoV-2 prototype and variants, as well as the Mosaic NP, we observed their binding signal to purified soluble hACE2 receptor using bio-layer interferometry. Previous studies have shown that the RBD of the B.1.1.7 variant has increased binding affinity to ACE2, while the RBD of B.1.351 the variant had a reduced binding signal compared with the wild-type RBD^16,40^. However, we found that the four HexaPro-bearing nanoparticles based on SARS-CoV-2 prototype and variants, as well as the Mosaic NP, exhibited equivalent binding affinity to the ACE2 receptor (Supplementary Table 1). We speculated that the self-assembled HexaPro-based nanoparticles in a repetitive array might have increased the antigen density and molecular size, resulting in a reduced rate of diffusion as well as a limited tendency for mass-transport. To examine the protein stability after nanoparticle assembly, we detected the intrinsic protein fluorescence and static light scattering signal during sample heating to measure the protein thermostability of HexaPro or its variants attached to the nanoparticles (Supplementary Fig. 2). The nanoparticles exhibited similar thermostability to wild-type HexaPro, although the nanoparticles displaying the HexaPro of Gamma variant alone or in combination with other HexaPro variants were more prone to aggregation. In addition, we also observed that the Gamma NP and Mosaic NP had an additionally reported Tm at about 45 °C and reported Tagg at a similar temperature, suggested that there was an extra unfolding region for Gamma spike protein and that the unfolding would cause protein aggregation (Supplementary Fig. 2).

### Immunogenicity of quadrivalent mosaic SARS-CoV-2 HexaPro-based nanoparticle vaccines in mice

**Fig. 2:**
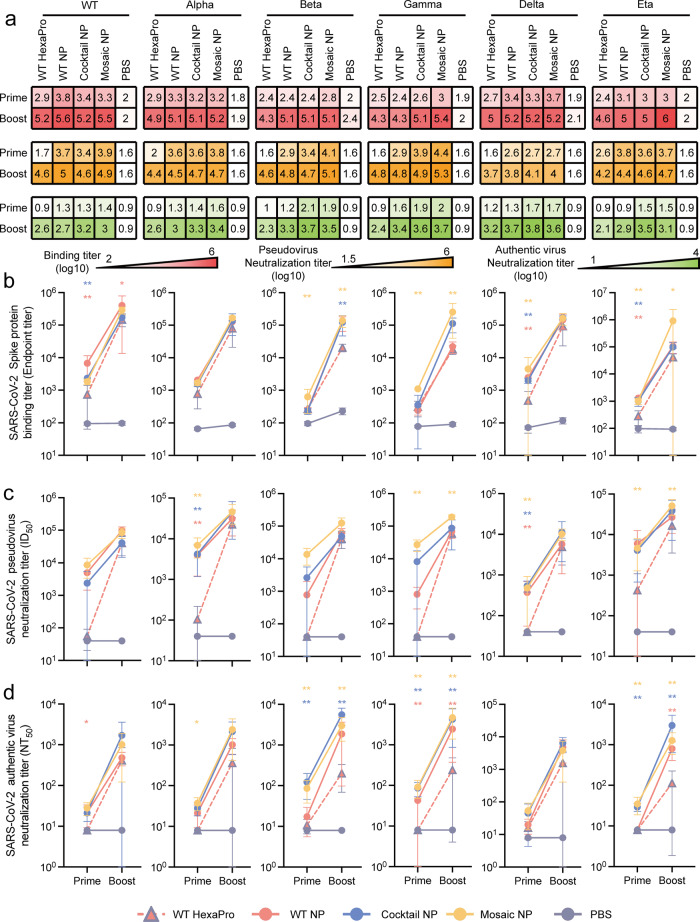
SARS-CoV-2 HexaPro-bearing immunogens induced a potent humoral immune response against SARS-CoV-2 prototype and circulating variants in mice. In all, 5 µg SARS-CoV-2 prototype HexaPro (*n* = 6), 6.5 µg SARS-CoV-2 HexaPro-based nanoparticle vaccine (including WT NP (*n* = 6), Cocktail NP (*n* = 6), Mosaic NP (*n* = 6) or PBS (*n* = 5) formulated with MF59 adjuvant were administered to the BALB/c mice via subcutaneous route at weeks 0 and 3. Sera were harvested at 2 weeks after primary and booster immunizations for ELISA binding and neutralization assay. **a** Summary of ELISA binding (**b**), pseudovirus (**c**), and authentic virus (**d**) neutralizing antibody titers. The ELISA binding, pseudovirus and authentic virus neutralization antibody titers elicited by HexaPro-bearing immunogens in mice after one and two vaccinations present as a heatmap colored in red, orange, and green, respectively. A deeper red, orange, and green represents a higher binding antibody titer, pseudovirus neutralization antibody titer and authentic virus neutralization antibody titer of sera from HexaPro-bearing immunogens-immunized mice, respectively. **b** HexaPro-specific endpoint binding titers of HexaPro-based immunogen-elicited sera in mice at weeks 2 and 5 against wild-type SARS-CoV-2 and a panel of 5 variants measured by ELISA. **c** Pseudovirus neutralization titers of HexaPro-based immunogen-elicited sera in mice at weeks 2 and 5 against wild-type SARS-CoV-2 and a panel of 5 variants. ID_50,_ reciprocal serum half-maximal neutralization in a pseudovirus-based neutralization assay. **d** Authentic virus neutralization titers of HexaPro-based immunogen-elicited sera in mice at weeks 2 and 5 against the ancestral strain and a panel of 5 variant SARS-CoV-2 strains. NT_50_, reciprocal serum half-maximal neutralization in the cytopathic effect-based neutralization assay. In b, c, and d, the dots indicate log_10_ (mean ± SD) titers. Statistical significance of each group was compared and analyzed using the two-tailed Mann–Whitney *U* test (*p < 0.05, **p < 0.01). Source data are provided as a Source Data file.

To characterize the humoral immune response to soluble Mosaic NP, BALB/c mice were immunized with either 5 µg of soluble WT HexaPro of SARS-CoV-2 prototype, 6.5 µg of HexaPro-based nanoparticles (WT NP, Cocktail NP, and Mosaic NP, equimolar amounts of WT HexaPro) of SARS-CoV-2, or PBS in the presence of an oil-in-water emulsion MF59-like adjuvant at days 0 and 21. Because of its safety and good tolerance profile in humans, MF59 adjuvant has been widely used to increase the immunogenicity and efficacy of vaccines against cytomegalovirus (e.g., NCT00133497), influenza virus (e.g., NCT03314662) and HIV (e.g., NCT00002204 and NCT03122223) in human clinical trials. Moreover, it was licensed for seasonal and pandemic influenza vaccines. After prime and boost administration, the binding antibody titers in the sera were determined by ELISA against HexaPro of SARS-CoV-2 wild type and a panel of 5 variants (including Alpha, Beta, Gamma, Delta, and Eta). As expected, sera from mice that received PBS formulated with MF59-like adjuvant contained almost no binding antibodies 2 weeks after the first and second dose (Fig. 2a, b). After the first vaccination, the levels of binding antibodies to wild-type HexaPro were higher in mice immunized with purified WT NP than in those immunized with purified trimeric WT HexaPro (ELISA median endpoint binding titer of 10^3.7 ± 0.4^ versus 10^2.7 ± 0.4^). These results were consistent with an increased humoral immune response against immunogens displayed on the surface of nanoparticles versus soluble immunogens^25,31,41^. By contrast, the levels of binding antibodies against SARS-CoV-2 HexaPro variants, including Beta, Gamma, Delta, and Eta, were higher in mice immunized with purified Mosaic NP than in those immunized with purified WT HexaPro (ELISA median endpoint binding titer of 10^2.8 ± 0.3^ versus 10^2.4 ± 0.1^, 10^3.0 ± 0.1^ versus 10^2.5 ± 0.2^, 10^3.5 ± 0.4^ versus 10^2.6 ± 0.3^, 10^3.0 ± 0.1^ versus 10^2.4 ± 0.2^, respectively). Two weeks after the second vaccination, the titers of antibodies specific for SARS-CoV-2 HexaPro wild type and variants elicited by all four designed vaccines were substantially increased (Fig. 2a, b). Immunization with Mosaic NPs elicited specific IgG titers against wild type, Alpha, Beta, Gamma, Delta and Eta HexaPro (median endpoint binding titers of 10^5.5 ± 0.1^, 10^5.2 ± 0.1^, 10^5.1 ± 0.2^, 10^5.4 ± 0.3^, 10^5.2 ± 0.2^, 10^6.0^^ ± 0.7^, respectively) that were 2- to 12.6-fold higher than those in mice immunized with WT HexaPro (10^5.2 ± 0.2^, 10^4.9 ± 0.4^, 10^4.3 ± 0.1^, 10^4.3 ± 0.1^, 10^5.0^^ ± 0.4^, 10^4.6 ± 0.4^, respectively). In addition, immunization with Cocktail NPs or Mosaic NPs elicited an equivalent or superior binding antibody response to the five HexaPro variants apart from wild-type HexaPro compared to those immunized with WT NPs (Fig. 2a, b), suggesting that homotypic HexaPro-based mosaic nanoparticle vaccines potentially increased the breadth and magnitude of the humoral immune response.

The neutralizing activity of sera induced by designed HexaPro-based nanoparticle immunogens was next assessed using a SARS-CoV-2 pseudotyped virus panel and a CPE-based authentic virus assay. As expected, sera from PBS-treated mice did not exhibit a neutralizing antibody response throughout the vaccination period (Fig. 2a, c). Two weeks after the first vaccination, the reciprocal serum half-maximal neutralization (ID_50_) titers in HEK293T-hACE2 cells were 7.9- to 631-fold higher in the mice that received Mosaic NP than in those that received soluble WT HexaPro against a panel of homotypic and heterotypic SARS-CoV-2 pseudovirions (prototype, Alpha, Beta, Gamma, Delta, and Eta; 10^3.9 ± 0.3^ versus 10^1.7 ± 0.2^, 10^3.8 ± 0.3^ versus 10^2^, 10^4.1 ± 0.2^ versus 10^1.6^, 10^4.4 ± 0.2^ versus 10^1.6^, 10^2.7 ± 0.4^ versus 10^1.6^, 10^3.7 ± 0.5^ versus 10^2.6 ± 0.8^, respectively). However, no significant difference was observed in matched or mismatched ID_50_ titers for mice that received WT NP versus Cocktail NP or Mosaic NP (Fig. 2a, b). Although the neutralizing antibody titers of sera from 4 HexaPro-based vaccine candidate formulated with MF59-like adjuvant were further boosted after the second dose, there was no statistically significant advantage in the ID_50_ titers between six SARS-CoV-2 pseudovirions except the Gamma and Eta variants (Fig. 2a, b), suggesting that the vaccine-induced antibody response may tend towards saturation.

The reciprocal serum-neutralization titer (NT_50_) against authentic virions of the SARS-CoV-2 variants (including Beta, Gamma and Eta) increased 4- to 20-fold in mice immunized with Mosaic NP compared to those immunized with soluble trimeric WT HexaPro after the prime and boost vaccination (Fig. 2a, d). Consistent with the results obtained from the ELISA and pseudotyped virus assay, immunization with Cocktail NP or Mosaic NP elicited an equivalent or superior neutralizing antibody response against authentic virus of SARS-CoV-2 prototype and two variants (Alpha and Delta) compared to those immunization with WT HexaPro over the whole immunization period (Fig. 2a, d). More importantly, the neutralization antibody response in mice triggered by Cocktail NP and Mosaic NP against the circulating SARS-CoV-2 VOCs and emerging VUMs remained at a relatively high and stable level according to the CPE-based authentic virus neutralization assay (Fig. 2a, d). These results indicated that in-silico rationally designed quadrivalent mosaic nanoparticle vaccines can effectively elicit high titers of neutralizing antibodies against both emerging and circulating VOCs and other emerging variants.

### Immunogenicity of quadrivalent mosaic SARS-CoV-2 HexaPro-based nanoparticle vaccines in cynomolgus macaques

**Fig. 3:**
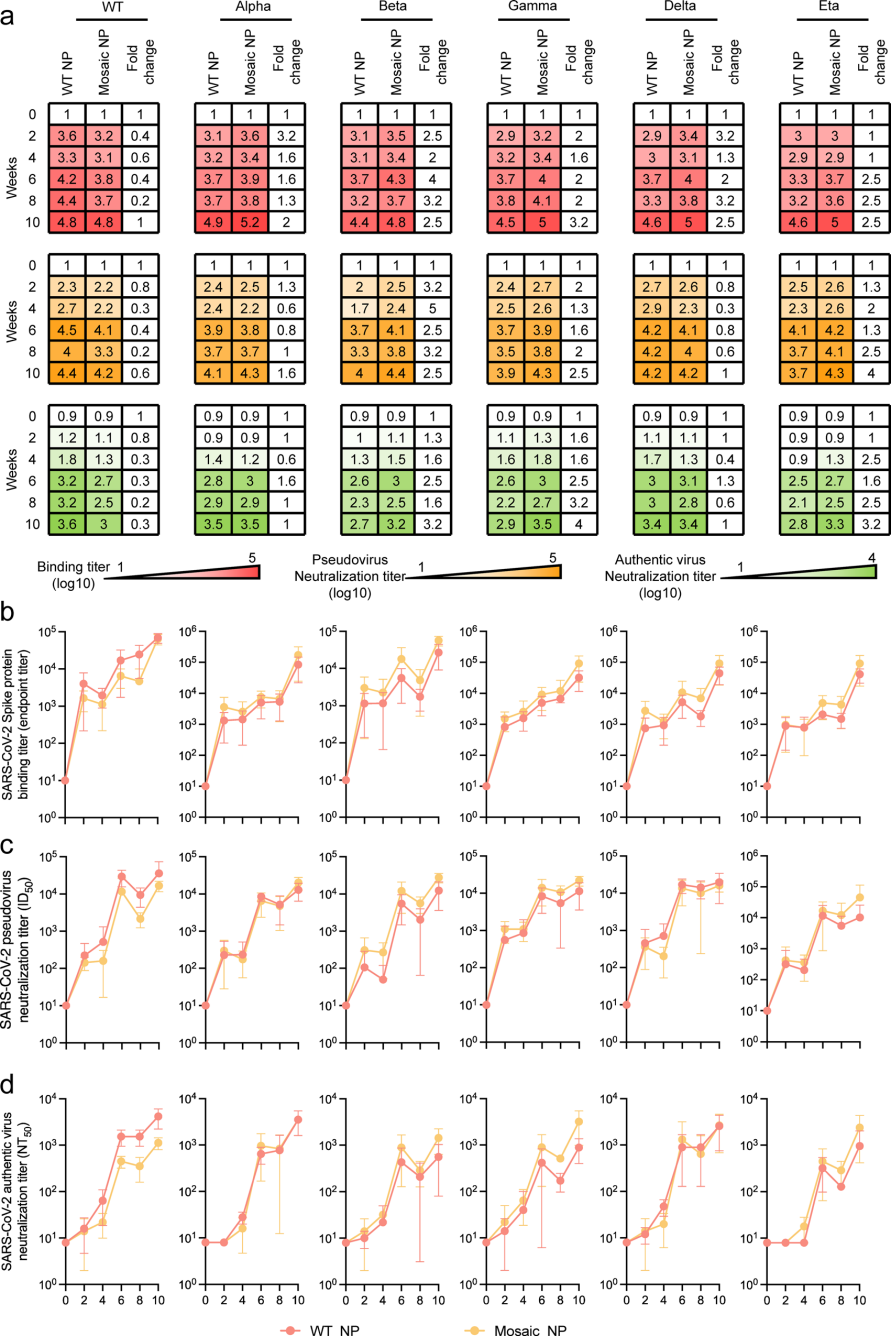
SARS-CoV-2 HexaPro-bearing immunogens induced a potent humoral immune response against SARS-CoV-2 prototype and circulating variants in cynomolgus macaques. **a** Summary of ELISA binding (**b**), pseudovirus (**c**), and authentic virus (**d**) neutralizing antibody titers. The binding, pseudovirus and authentic virus neutralization antibody titers of HexaPro-bearing immunogen-elicited sera from cynomolgus macaques following each vaccination 4 weeks apart present as a heatmap colored in red, orange and green, respectively. A deeper red, orange and green represents a higher binding antibody titer, pseudovirus neutralization antibody titer and authentic virus neutralization antibody titer of sera from HexaPro-bearing immunogens-immunized mice, respectively. **b** HexaPro-specific endpoint binding titers of HexaPro-based immunogen-elicited sera in cynomolgus macaques following each vaccination 4 weeks apart against wild-type SARS-CoV-2 and variants measured by ELISA. **c** Pseudovirus neutralization titers of HexaPro-based immunogen-elicited sera from cynomolgus macaques following each vaccination 4 weeks apart against wild-type SARS-CoV-2 and a panel of 5 variants. ID_50_, reciprocal serum half-maximal neutralization in the pseudovirus-based neutralization assay. **d** Authentic virus neutralization titers of HexaPro-based immunogen-elicited sera from cynomolgus macaques following each vaccination 4 weeks apart against ancestral SARS-CoV-2 and a panel of 5 variant strains. NT_50_, reciprocal serum half-maximal neutralization in the cytopathic effect-based neutralization assay. In **b**–**d**, the dots indicate log_10_ (mean ± SD) titers. Each group contained four cynomolgus macaques. The statistical significance of each group was compared and analyzed using the two-tailed Mann–Whitney *U* test. Source data are provided as a Source Data file.

Due to the potency and breadth of cross-neutralizing antibodies as well as the efficacy of protection induced by mosaic quadrivalent SARS-CoV-2 HexaPro-based nanoparticle vaccines in mice, we further evaluated their immunogenicity in cynomolgus macaques, a species that is immunologically and physiologically closer to humans. No neutralizing antibodies against a panel of SARS-CoV-2 pseudotyped viruses were detected in any cynomolgus macaques prior to immunization. Eight cynomolgus macaques were randomly divided into two groups (*n* = 4) and intramuscularly immunized with either a total of 65 μg of WT NP or Mosaic NP immunogen formulated with MF59-like adjuvant in quadricep at weeks 0, 4, and 8. Serum samples were collected fortnightly and heat-inactivated prior to immunological and virological experiments. We examined and compared the binding antibody titers of sera elicited by WT NP and Mosaic NP in cynomolgus macaques to vaccine-matched (wild type, Alpha, Beta, and Gamma variant) or mismatched (Delta and Eta variant) HexaPro. In comparison with WT NP, cynomolgus macaques exhibited Mosaic NP-elicited wild type HexaPro-specific binding antibody titers that were lower from week 2 to week 8 but tended to be equal at week 10. However, Mosaic NP induced higher binding antibody titers against a panel of HexaPro variants (including Alpha, Beta, Gamma, Delta, and Eta) than WT NP at all time points (Fig. 3a), although we did not observe a statistically significant difference in binding antibody levels for the aforementioned HexaPro when comparing WT NP and Mosaic NP.

Similarly, we also conducted HIV-based pseudotyped virus and CPE-based authentic virus neutralization assays to evaluate the ability of the non-human primate sera to neutralize a panel of SARS-CoV-2 viruses. Consistent with the results from mice, the median autologous ID_50_ and NT_50_ titers against SARS-CoV-2 prototype were lower in non-human primates immunized with Mosaic NP than in those immunized with WT NP during all 10 weeks of immunization, although the difference was not statistically significant (Fig. 3a, c, d). Sera elicited by Mosaic NP induced equivalent ID_50_ and NT_50_ titers against SARS-CoV-2 variants (Alpha and Delta) compared to those immunized with WT NP during the whole immunization period (Fig. 3a, c, d). However, the ID_50_ titers of Mosaic NP-induced sera against SARS-CoV-2 variant (Beta, Gamma and Eta) pseudoviruses was 1.3- to 5-fold higher than against WT NP-induced serum at all time points. Compared with WT NP, non-human primates vaccinated with Mosaic NP exhibited higher NT_50_ titers against homologous authentic SARS-CoV-2 virions comprising Beta, Gamma and Eta variants at all time points. In particular, the NT_50_ titers against authentic SARS-CoV-2 viruses comprising Beta, Gamma and Eta spikes were 3.2- to 4-fold higher in non-human primates immunized with Mosaic NP than in those immunized with WT NP at 2 weeks post second boost (10^3.2 ± 0.2^ versus 10^2.7 ± 0.3^, 10^3.5 ± 0.3^ versus 10^2.9 ± 0.2^, 10^3.3 ± 0.4^ versus 10^2.8 ± 0.4^, respectively) (Fig. 3a, d), although the difference was not statistically significant. In addition, we observed that the ID_50_ titers of the two groups against all SARS-CoV-2 pseudoviruses tested in this study increased over time following the first vaccination and retained a relatively high level 2 weeks after the second vaccination. However, they did not show a significant increase trend after the third immunization (Fig. 3a), indicating that strategies to reduce the number of vaccinations to economize the available vaccine doses might not compromise the resulting titer of neutralizing antibodies when using a HexaPro-based nanoparticle vaccine.

**Fig. 4:**
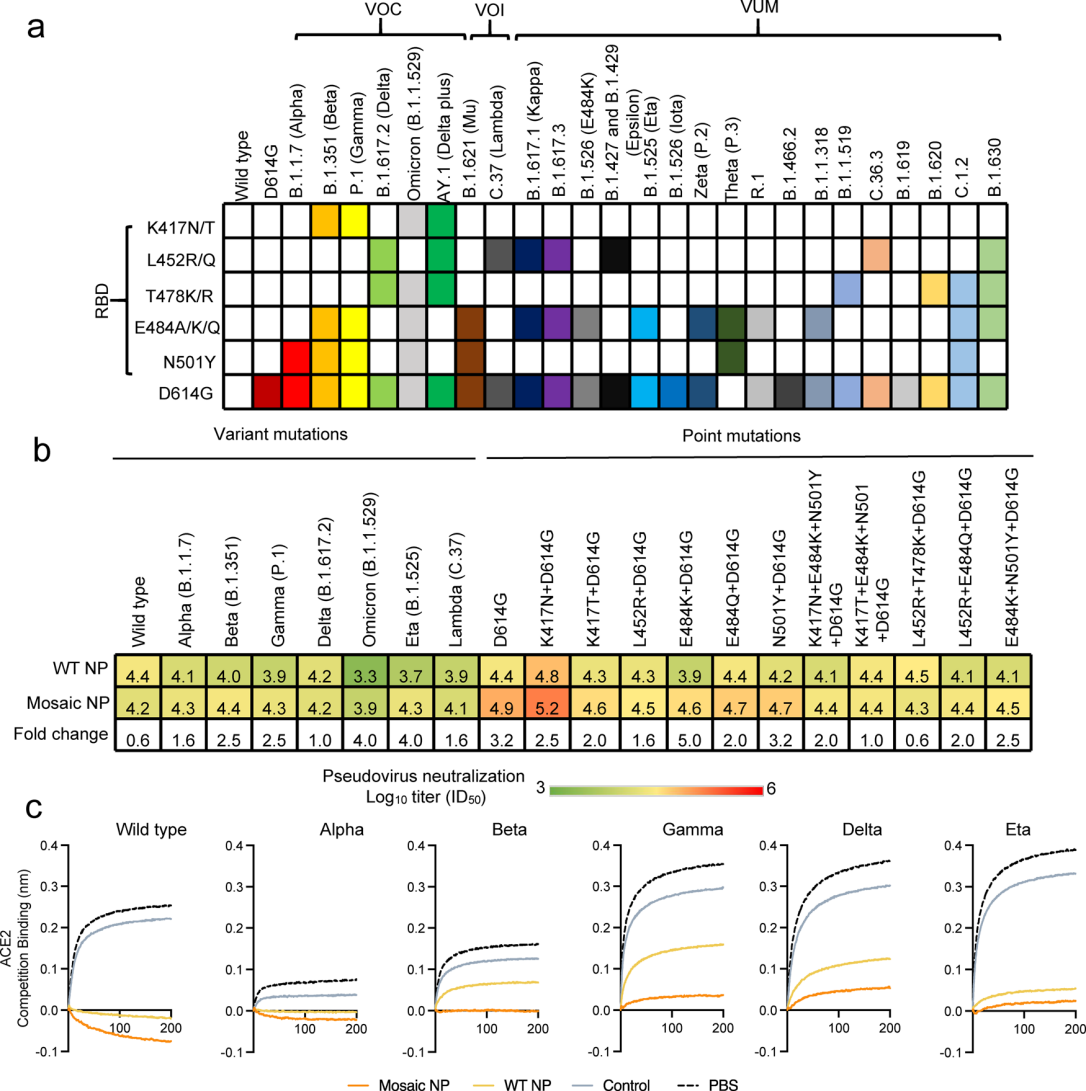
Quadrivalent mosaic SARS-CoV-2 nanoparticle vaccine-elicited potent and broad neutralization antibody responses in cynomolgus macaques against pseudoviruses with various mutations in the RBD of SARS-CoV-2. **a** RBD key residue substitutions that affect the neutralizing sensitivity of ancestral and variant SARS-CoV-2 isolates are colored. The RBD residue mutations of circulating SARS-CoV-2 variants were downloaded from the Global initiative on sharing all influenza data (GISAID) EpiCoV database. RBD receptor-binding domain, VOC Variant of Concern, VOI Variant of Interest, VUM Variants Under Monitoring. **b** Neutralization antibody titers of HexaPro-based SARS-CoV-2 nanoparticle vaccine-elicited sera 2 weeks after the second booster dose (*n* = 4 cynomolgus macaques in each group) against a panel of SARS-CoV-2 mutant pseudoviruses. The neutralization titers were expressed as log_10_ (means ± SD). The pseudoviruses neutralization titers were present as a heatmap colored in red-to-green gradient. A deeper red represents a higher pseudovirus neutralization activity, and a deeper green a lower pseudovirus neutralization activity. **c** BLI binding competition analysis with the hACE2 receptor of serum samples at peak potency collected from cynomolgus macaques that were vaccinated with HexaPro-based nanoparticle vaccines. Source data are provided as a Source Data file.

Apart from the SARS-CoV-2 prototype and variants, we also examined the breadth of neutralizing antibodies from cynomolgus macaques immunized with WT NP or Mosaic NP at peak potency (2 weeks after a third vaccination) against pandemic Omicron and Lambda variants and a panel of SARS-CoV-2 pseudoviruses harboring single or combinatorial key residue mutations in the RBD (K417N/T, L452R, T478K, E484K/Q, N501Y) from the circulating pango lineage (Fig. 4a). As shown in Fig. 4b, the Omicron and Lambda variants reduced the neutralization potency of sera elicited by WT NP (median ID_50_ titer of 10^3.3 ± 0.2^ and 10^3.9 ± 0.4^, respectively) was 20.0- and 5.0-fold lower compared to the SARS-CoV-2 prototype. However, the Omicron and Lambda variants did slightly reduce the neutralization potency of sera induced by Mosaic NP (median ID_50_ titer of 10^3.9 ± 0.1^ and 10^4.1 ± 0.4^, respectively), suggested that Mosaic NP-induced sera elicited broadly protective antibody responses to circulating SARS-CoV-2 variants. The aforementioned single residue RBD mutations were documented to affect the neutralization sensitivity to mAbs and sera from vaccinated individuals^42,43^. Previous sequence analyses have shown that the circulating D614G substitution variant has become the dominant isolate as the pandemic spread^13^, although it increased the susceptibility to neutralization by vaccinate-elicited sera and mAbs^12^. The D614G substitution altered the neutralization potency of sera elicited by WT NP (median ID_50_ titer of 10^4.4 ± 0.2^) and Mosaic NP (median ID_50_ titer of 10^4.9 ± 0.5^) was 1.0- to 5.0-fold higher compared to the SARS-CoV-2 prototype. Compared with SARS-CoV-2 wild-type Spike (D614G), four single residue RBD mutation (including K417N/T, L452R, E484Q, and N501Y) did not significantly affect the neutralization potency of sera induced by WT NP or Mosaic NP (Fig. 4b). For instance, the K417N substitution found in the B.1.351 and AY.1 lineages (median ID_50_ titer of 10^4.8 ± 0.2^ for WT NP and 10^5.2 ± 0.5^ for Mosaic NP), E484Q present in B.1.617.1, B.1.617.3, and B.1.630 (median ID_50_ titer of 10^4.4 ± 0.4^ for WT NP and 10^4.7 ± 0.2^ for Mosaic NP) increased it 2.5- (K417N) and 1.0-fold (E484Q) for WT NP, as well as increased it 2.0 (K417N) and reduced it 1.6-fold (484Q) for Mosaic NP. The N501Y mutation (present in B.1.1.7, B.1.351, P.1, B.1. 621, Theta and C.1.2) led to a 1.6-fold decrease in neutralization potency (median ID_50_ titer of 10^4.2 ± 0.1^ for WT NP and 10^4.7^ for Mosaic NP). The L452R substitution present in B.1.617.2, AY.1, B.1.617, B.1.617.3, B.1.427, B.1.429, C.36.3, and B.1.630 (median ID_50_ titer of 10^4.3 ± 0.2^ for WT NP and 10^4.5 ± 0.2 ^for Mosaic NP) elicited 1.3-fold and 2.5-fold decrease in neutralization potency compared to SARS-CoV-2 wild type (D614G). However, the E484K substitution present in B.1.351, P.1, B.1.621, B.1.525, Zeta, Theta, R.1, B.1.1.318, C.1.2, and B.1.630 (median ID_50_ titer of 10^3.9 ± 0.3^ for WT NP and median ID_50_ titer of 10^4.6 ± 0.1^ for Mosaic NP) elicited 3.2-fold lower neutralization potency for WT NP and 2.0-fold lower neutralization potency for Mosaic NP than SARS-CoV-2 wild type (D614G), indicating that the E484K substitutions exhibited a certain level of resistance for WT NP. The combinatorial mutations in SARS-CoV-2 RBD, including K417N + E484K + N501Y, K417T + E484K + N501Y, L452R + T478K, L452R + E484Q, E484K + N501Y, present in several SARS-CoV-2 variants (e.g. B.1.1.7, B.1.351, and B.1.617.2), obviously reduced the neutralization potency of sera from non-human primates vaccinated with WT NP or Mosaic NP compared with SARS-CoV-2 prototype (D614G) (Fig. 4b), but there still remained a very high neutralization activity of sera elicited by multivalent HexaPro displayed on the nanoparticles.

The genus betacoronavirus (SARS-CoV and MERS-CoV) includes bat coronaviruses (such as RATG13) and common human coronaviruses (including OC43, HKU1, NL63, and 229E) that spread with epidemic features in animals and humans and still pose a serious threat to human health. Therefore, we also evaluated the neutralization potency of sera from non-human primates immunized with WT NP or Mosaic NP against SARS-CoV, MERS-CoV, HCoV-OC43, HCoV-NL63, HCoV-229E, and RATG13 pseudoviruses. Following 2 weeks after the third immunization, non-human primates vaccinated with Mosaic NP exhibited higher levels of neutralization antibodies against all SARS-related pseudoviruses than those vaccinated with WT NP (Supplementary Fig. 3). To further investigate the magnitude of binding antibodies elicited by the HexaPro-based nanoparticle vaccine, we performed competitive BLI analysis with hACE2, which binds to the receptor-binding domain of spike protein. At a 1000-fold dilution, serum from cynomolgus macaques vaccinated with Mosaic NP adjuvanted with MF59 was more competitive than the ACE2 receptor for binding to wild-type, Alpha, Beta, and Gamma variants but not for HexaPro trimer of Delta and Eta variants than those vaccinated with WT NP (Fig. 4c). In general, these results demonstrated that sera from cynomolgus macaques vaccinated with Mosaic NP exhibited higher and broader binding and neutralization potency than those elicited by WT NP.

### Mosaic HexaPro-based nanoparticle vaccines protect mice against homotypic SARS-CoV-2 challenge

**Fig. 5:**
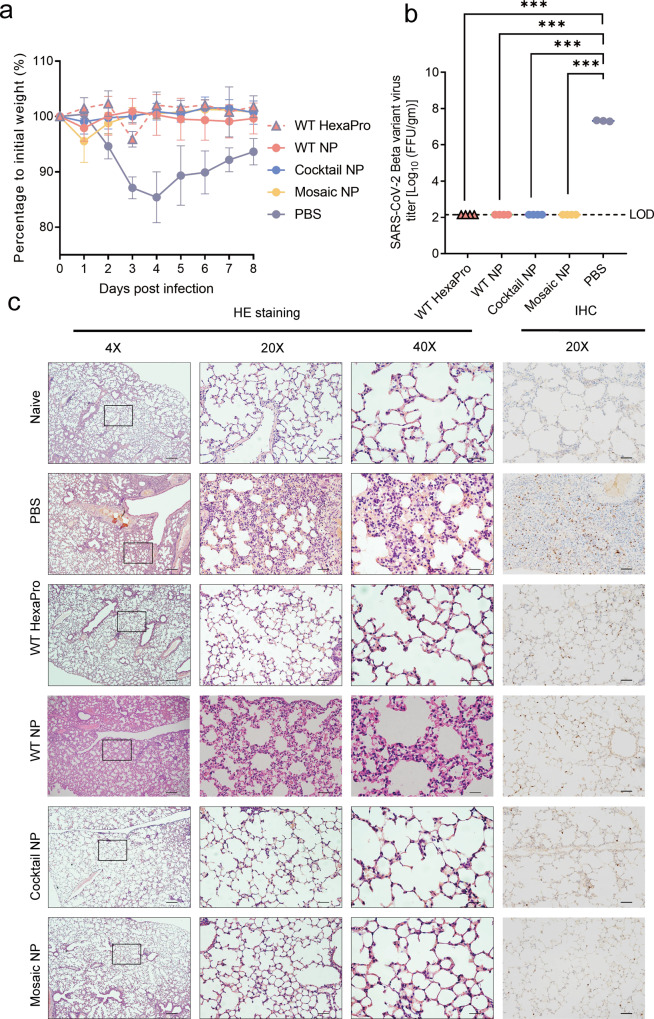
Protective efficacy of HexaPro-bearing immunogens in mice following challenge with SARS-CoV-2 B.1.351 variant in vivo. **a**–**c** six-week-old male BALB/c mice were subcutaneously immunized with an equivalent amount of HexaPro-based immunogen (equal to 5 µg HexaPro) at weeks 0 and 3. At 4 weeks after the second vaccination, the mice were intranasally inoculated with 2 × 10^6^ PFU/ml SARS-CoV-2 B.1.351 variant authentic virus. Lung tissues were collected for virus titer qualification and clinicopathological analysis. **a** Body weight changes of mice (*n* = 4 mice in HexaPro-based-vaccinated group, *n* = 2 in PBS-treated group) after infection with SARS-CoV-2 B.1.351 variant. The body weight of each mouse was recorded daily for 8 days. **b** Virus titers of lung tissues from SARS-CoV-2 B.1.351 variant-challenged mice (*n* = 4 mice in HexaPro-based-vaccinated group, *n* = 3 in PBS-treated group) at 2 days post challenge. LOD limit of detection. Statistical significance was determined by two-tailed unpaired t test. WT HexaPro, WT NP, Cocktail NP, and Mosaic NP vs PBS, ***p = 0.0001. **c** Immunohistological analysis of lung tissues from SARS-CoV-2 B.1.351 variant-challenged mice (*n* = 2 mice in each experimental group) at 4 days post challenge. Hematoxylin and eosin staining (HE, left) and immunohistochemistry (IHC, right) microscopic images are shown in the figure at magnification. Scale bar for H&E, 250 μm (left); 50 μm (middle); 25 μm (right), Scale bar for IHC, 50 μm. In **a** and **b**, the data were expressed and plotted as means ± SD. Source data are provided as a Source Data file.

To investigate the neutralization potency of sera elicited by quadrivalent mosaic SARS-CoV-2 HexaPro-based nanoparticle prototype and variant virus in vivo, mice were initially inoculated with authentic SARS-CoV-2 B.1.351 variant virus. Previous studies have reported that BALB/c mice were susceptible to infection with the SARS-CoV-2 B.1.351 variant without Ad5-hACE2 transduction and developed typical bronchopneumonia with viral replication^44^. The body weight of PBS-treated mice was decreased gradually at day 1 post infection, and reached to peak up to 14.6% loss at day 4 post infection, and then showed a recovery trend. However, sera elicited by immunization with HexaPro-based immunogens almost completely prevented SARS-CoV-2 B.1.351 variant-caused body weight loss in mice during the 8-day observation period (Fig. 5a). Two days after challenge, the lungs were harvested for viral burden and cytokine analysis. No infectious virions were detected in the lung homogenates collected from mice immunized with soluble trimeric HexaPro and HexaPro-based nanoparticle vaccine using focus forming assay (FFA) (Fig. 5b). Compared to the PBS-treated control group, the number of Open Reading Frame 1ab (*ORF1ab)* gene copies in the lungs of mice immunized with WT HexaPro, WT NP, Cocktail NP, and Mosaic NP were decreased 10^4.0^, 10^4.4^, 10^4.4^, and 10^4.7^-fold at day 2 post infection, respectively. The number of Nucleocapsid (*N*) gene copies in four HexaPro-based vaccine-immunized groups is analogous to the number of *ORF1ab* gene copies (Supplementary Fig. 4a). COVID-19 is characterized by a cytokine storm. As an indication of disease severity, we measured the mRNA expression levels of IFN-stimulated genes (*IFIT1*, *IFIT3*, *ISG15*, and *MX2*), pro-inflammatory cytokines (*IL6*, *IL10*), and chemokines (*CXCL10*, *CCL2*) that are correlated with COVID-19 exacerbation. Quantitative RT-PCR profiles showed that the expression of inflammatory genes and cytokine in the lung following SARS-CoV-2 B.1.351 variant challenge in each group of mice that received the HexaPro-based vaccine, was significantly lower than in mice that received PBS (Supplementary Fig. 4b). We next evaluated the efficacy of HexaPro-based nanoparticle vaccine against SARS-CoV-2-induced pulmonary pathology. Histopathological and immunohistochemical inspection of lung tissues from PBS-treated mice showed accumulation of inflammatory cells in capillaries resulting in septal thickening, collapsed alveolar spaces, and resident large quantities of viral antigens. By contrast, no or mild lung pathology and viral antigen abundance was observed in the lungs of mice vaccinated with any of the HexaPro-based vaccines (Fig. 5c). In addition, mice that received a second dose of vaccine after 4 weeks and then transduced intranasally with Ad5 stably expressing human ACE2 (Ad5-hACE2), were inoculated with SARS-CoV-2 prototype after five days of transduction. Consistent with the protective efficacy against SARS-CoV-2 B.1.351 variant infection, mice vaccinated with HexaPro-based immunogens were also protected against SARS-CoV-2 wild-type infection (Supplementary Figs. 5 and 6). Overall, these experiments indicated that HexaPro-based immunogens, can effectively protect mice from SARS-COV-2 prototype and B.1.351 variant strain infection.

## Discussion

So far, several vaccines based on SARS-CoV-2 spike protein were approved and given emergency use authorizations by national regulators around the world and the World Health Organization (WHO), including mRNA-1273, BNT162b2, Ad26.COV2.S, AZD1222, and NVX-COV2373^17^. Phase III clinical trials have shown that after the second dose, the efficacy of these licensed vaccines against ancestral SARS-CoV-2 infection was 66.1 to 95% in preventing symptomatic COVID-19 infection^9,17,45^. However, as SARS-CoV-2 spread and caused a pandemic, new variants, including VOCs, emerged and brought new challenges for global containment of COVID-19, due to their reduced sensitivity to current vaccines based on wild-type antigens^1,15,18,20,21,43^. Studies have shown that vaccine-induced or convalescent sera manifested reduced neutralization activity against SARS-CoV-2 variants, especially the Beta (B.1.351)^1,18,43^ and Omicron (B.1.1.529) variant^6,7,46^, indicating that current vaccine design based on wild-type antigen may not elicit sufficient protective antibodies against these variants. Therefore, the value of using mutant spike antigens as vaccine candidates against SARS-CoV-2 variant strains deserves further investigation.

A recent study using an mRNA vaccine based on the Beta variant spike protein found that such design could elicit higher neutralizing antibody titers against Beta variant infection in comparison to the wild-type spike in non-human primates, showing the great potential of mutant spike proteins in designing variant-specific vaccines^47^. However, there is still a lack of systematic investigation of spike protein variants as a generalized strategy for the development of vaccines against emerging VOCs and other variants. In our study, the quadrivalent nanoparticle comprising spike proteins from the wild type and three variants (Alpha, Beta, and Gamma) strains could elicit relatively higher neutralizing antibody titers against pseudovirus or authentic virus infection of the three SARS-CoV-2 variants included as antigens, and even another two not included variants (Delta and Eta) in both mouse and non-human primate models (Figs. 2 and 3). This result supported the idea of using variant immunogens in combination with variant strains during vaccine design. However, the difference between wild-type and cocktail or mosaic nanoparticles was mostly not statistically significant, especially in non-human primate immunization and mouse challenge experiments, indicating that the divergence in spike protein antigen at residue-level may not be sufficient to generate significantly heterogenous antibody responses against various strains.

However, whether such differences without statistical significance could bring clinical benefit remained uncertain. Studies of vaccine effectiveness against SARS-CoV-2 VOCs revealed that the decrease of neutralization potency for various VOCs varied from several times to a few percentage points^9,19^, and such differences could be influenced by the number of experimental replications, vaccination dose of HexaPro-based immunogens or the studied population. In our study, we found that mosaic NPs could elicit higher neutralization titers than WT NP, with an increase of 0 to 5-fold. Although the improvement of antibody titers did not manifest statistical significance due to the limited number of subjects, such superiority was still widely observed in the assays on the five variants strain. In addition, consistent with mosaic RBD-NP^23^, mice and non-human primates immunized with a low dose of HexaPro-based nanoparticle vaccine will help to optimize the antigen design. Therefore, there was reason for us to believe that the enhanced neutralization ability brought by the introduction of variant antigens in nanoparticle design was practical and deserved further investigation in larger animal experiments or clinical studies.

With the rapid spread of SARS-CoV-2 variants, such as B.1.617.2 (Delta) and B.1.1.529 (Omicron) variants, low protection rate and increasing number of cases of breakthrough infection after vaccinated with the licensed COVID-19 vaccine were reported^48,49^. Strategies such as extra booster dose vaccination, immunogen optimization, and multivalent vaccine design with broad target antigen may be the viable solutions for immune escape of SARS-CoV-2 variants. Previous studies showed that mice immunized with mosaic SARS-related RBD-based nanoparticle vaccine could be elicited broadly antibody response to SARS-CoV-2 and SARS-related emergent zoonotic coronaviruses^23,50^. And rhesus macaques vaccinated with a D614G/B.1.351 bivalent RBD-based nanoparticle vaccine potentially induced cross-neutralizing antibodies for SARS-CoV-2 variants^51^. In this study, we developed a quadrivalent mosaic nanoparticle vaccine displaying the HexaPro spike proteins of SARS-CoV-2 prototype and VOCs (Mosaic NP) with potent efficacy of inducing robust neutralizing antibody responses against SARS-CoV-2 prototype and circulating variants in mice and non-human primates. Given that SARS-CoV-2 Omicron variant was dominant strain in global circulation and that Mosaic NP exhibited the capability of inducing robust neutralizing antibody response against Omicron variant compared to WT NP (Fig. 4b), our results indicated a direction to develop a multivalent nanoparticle vaccine targeting multiple strains of SARS-CoV-2.

We further put effort in studying the value of in-situ mosaic arrangement of the four different spike proteins on the nanoparticle surface, instead of simple mixing of four different spike protein nanoparticles (Cocktail NP). In the mouse immunization assay, we observed that mosaic NP maintained a slight but stable advantage over the Cocktail NP in both total binding and neutralizing antibody titers, suggesting that the multivalent mosaic nanoparticle design had stronger potency in eliciting antibodies against multiple SARS-CoV-2 variants than a mixture of monovalent nanoparticles. Consistent with other researcher reports^52^, we speculated that such a strategy could present several different spike variants to a single B cell due to the mosaic design, and could more efficiently stimulate those that express B cell receptors able to effectively recognize different spike protein variants, leading to the generation of antibodies with broader and stronger neutralizing capability against SARS-CoV-2 variants. In comparison, the Cocktail NP or mixture of spike protein variants could not achieve such presentation due to the innate deficiency of separate distribution of antigens on different nanoparticles. Therefore, it might be possible that the mosaic nanoparticle could be more helpful in eliciting neutralizing antibodies with broad effectiveness against a wide range of SARS-CoV-2 variants, beyond its original use as vaccine candidate.

## Methods

### Cell culture

Adherent cells, including HEK293T (ATCC, CRL-3216), Vero E6 (ATCC, CRL-1586), and Vero (ATCC, CCL-81) were cultured in Dulbecco’s minimal essential medium (DMEM, ThermoFisher Scientific, Cat#11995065) supplemented with 25 mM D-glucose, 1 mM sodium pyruvate, 1×non-essential amino acids, 10% (v/v) heat-inactivated fetal bovine serum (FBS, ExCell Bio, Cat#FSP500) and 1% penicillin–streptomycin (GIBCO, Cat#15140122) at 37 °C in 5% CO_2_. The hACE2-HEK293T cell line was derived from the HEK293T cell line stably expressing human angiotensin-converting enzyme 2 (hACE2) by infection with a lentiviral vector that encodes the full-length hACE2 gene. Suspension Expi293F^TM^ cells (Thermo Fisher Scientific, Cat#A14527) were maintained in Union 293 medium (Union-Biotech, Cat#UP1000) supplemented with 1% penicillin–streptomycin (ThermoFisher Scientific, Cat#15140122) at 37 °C and 5% CO_2_ with shaking at 120 rpm. All cell lines listed above were tested negative for mycoplasma contamination by Polymerase Chain Reaction (PCR) on a weekly basis.

### Viruses

The two SARS-CoV-2 prototype strains, viz. SARS-CoV-2/human/CHN/IQTC01/2020 (GenBank accession ID: MT123290.1) and 2020XN4276 (GISAID accession ID: EPI_ISL_413859), were isolated from COVID-19 patients and obtained from the Guangzhou Medical University and Guangdong Provincial Center for Disease Control and Prevention. Five SARS-CoV-2 variant strains, viz. 2021A-XG04123 (Lineage: P1, WHO label: Gamma), 2021A-XG02292 (Lineage: B.1.1.7, WHO label: Alpha), 20SF18530 (Lineage: B.1.351, WHO label: Beta, GISAID accession ID: EPI_ISL_2536954), 2021K-XG0186 (Lineage: B.1.617.2, WHO label: Delta), and 2021A-XG02275 (Lineage: B.1.525, WHO label: Eta), were obtained from throat swabs in confirmed imported COVID-19 cases from outside China. All viruses used in this study were passaged three times in Vero-E6 cells and then purified by plaque assay^53^. Virus titers were determined via the median tissue culture infectious dose or plaque-forming assay. In this study, all authentic virus experiments including virus isolation, titration, neutralization, and challenge were conducted in an approved biosafety level-3 (BSL-3) facility.

### Gene synthesis and plasmid construction

Plasmids encoding the I53-50A1 trimer and I53-50B.4PT1 pentamer fused with a C-terminal octahistidine tag (HHHHHHHH) were codon optimized for expression in *Escherichia coli* cells, synthesized by GenScript, and introduced into the pET28b prokaryotic expression vector using the *Nco*I and *Hin*dIII restriction enzyme sites. The prototypic HexaPro trimer (residues 16-1138) (GISAID accession ID: EPI_ISL_413859), and five additional variants, viz. England/MILK-9E05B3/2020 (Lineage: B.1.1.7; GISAID accession ID: EPI_ISL_601443), South Africa/NHLS-UCT-GS-1067/2020 (Lineage: B.1.351, GISAID accession ID: EPI_ISL_700428), Japan/IC-0561/2021 (Lineage: P.1; GISAID accession ID: EPI_ISL_792680), hCoV-19/Nigeria/CV844/2021 (Lineage: B.1.525; GISAID accession ID: EPI_ISL_1235642), and hCoV-19/India/MP-NCDC-2509230/2020 (Lineage: B.1.617.2; GISAID accession ID: EPI_ISL_2461258), were codon-optimized for expression in Expi293F^TM^ cells, synthesized by GenScript, and cloned using the *Bam*HI restriction enzyme into the VRC8405 mammalian expression vector with an N-terminal Kozak sequence and tissue plasminogen activator signal peptide (MDAMKRGLCCVLLLCGAVFVSPSAS), and a C-terminal flexible linker (GSGG), T4 foldon trimerization domain (GYIPEAPRDGQAYVRKDGEWVLLSTFL), human rhinovirus 3C protease recognition site (GSRSLEVLFQGP) and octa-histidine tag (GSGHHHHHHHH). The plasmid expressing SARS-CoV-2 HexaPro contained 6 proline substitutions: F817P, A892P, A899P, A942P, K986P, and V987P, and a GSAS amino acid sequence substituted at the furin cleavage site (residues 682-685) as previously described^37^. To construct the fusion plasmid expressing HexaPro-foldon-I53-50 A1 for both SARS-CoV-2 wild type and other variants, the sic_axle docking protocol^54^ was used to compute the distance between HexaPro-foldon and the I53-50A1 trimer. The HexaPro-foldon was connected to the N-terminus of the I53-50A1 trimeric components using a flexible linker containing 16 glycine and serine residues (GGSGGSGSGGSGGSGS) and a rigid linker (EKAAKAEEAARK) based on the docking model results. The four HexaPro-foldon-I53-50 A1 fusion plasmids were synthesized by GenScript and cloned into the VRC8405 vector. The monomeric hACE2 (residue 19-615) construct was created as previously reported^31^. The single or combinatorial residue substitutions in the RBD of spike protein were downloaded from Centers for Disease Control and Prevention (https://www.cdc.gov/coronavirus/2019-ncov/variants/variant-info.html), and introduced into the pCMV14 vector using Mut Express II Fast Mutagenesis Kit V2 (Vazyme, Cat#C214-02) following the manufacturer’s instructions. A panel of expression plasmids encoding the spike protein of SARS-CoV-2 prototype and variants (Lineages: B.1.1.7, B.1.351, P.1, B.1.525, B.1.617.2, B.1.525, and C.37) with a 19-residue truncation at the C-terminus for SARS-CoV-2 pseudovirus production was constructed by Gibson assembly. Gene fragments encoding HCoV-229E (GenBank: APT69883.1), MERS-CoV (GenBank: AFS88936.1), HCoV-NL63 (GenBank: APF29071.1), HCoV-OC43 (GenBank: AVR40344.1.), RATG13 (GenBank: QHR63300.2), and SARS-CoV (GenBank: AAP13567.1) spike glycoproteins with a 19-residue truncation at the C-terminus for the generation of pseudotyped viruses were similarly amplified and ligated into the pCMV14 vector by overlap extension PCR and Gibson assembly.

Plasmids expressing heavy and light chains of mAbs (REGN-10933, Regdanvimab, S2-E12, COVA1-16, S2-H14, S2-M11, CB6, IgG1-ab1, P2B-2F6, CR3022, COV2-2196, 4A8, Fab 2-15, and REGN-10987) from the coronavirus antibody database (http://opig.stats.ox.ac.uk/webapps/covabdab) were synthesized by GenScript and cloned into a mammalian cell expression vector.

### Transient transfection

The recombinant plasmid DNA of all HexaPro-foldon, HexaPro-foldon-I53-50A1 trimer, monomeric hACE2 and 14 mAbs as described above was extracted from bacterial culture of transformed DH5a competent cells in Terrific Broth medium according to manufacturer’s standard protocol (Macherey-Nagel, NucleoBond® Xtra Maxi, Cat#740414.50). Expi293F^TM^ cells were grown in Union 293 medium, diluted to a density of 1.0×10^6^ cells per mL, and then transiently transfected with 1:3 (v/v) containing 1 mg recombinant plasmid:1 mg/ml PEI-MAX (Polyscience, Cat#24765) in Union 293 medium. After 5 days, the cell cultures containing targeted proteins were centrifuged to remove cell debris at 15,000×*g* for 2 h, and then filtered using a 0.22 µm pore-size vacuum-driven filter.

### Recombinant protein expression and purification

For HexaPro trimer and HexaPro-foldon-I53-50A1, the clarified supernatant was loaded onto a Ni Sepharose excel resin (Cytiva, Cat#17371202) column equilibrated with composition buffer A (50 mM HEPES pH 8.0, 300 mM NaCl, 5% glycerol, 0.5% (w/v) 3-[(3-cholamidopropyl)dimethylammonio]-1-propanesulfonate (CHAPS) and 0.02% NaN_3_). After gravity-fed loading, the column was washed with 10 column volumes of buffer A supplemented with 30 mM imidazole, and then eluted with 10 column volumes of buffer A supplemented with 500 mM imidazole, after which the protein was further purified by size exclusion chromatography on a Superdex 200 Increase 10/300 GL column (Cytiva, Cat#28-9909-44) equilibrated with composition buffer A. The molecular weight and purity of the elution fractions were tested using sodium dodecyl sulfate polyacrylamide gel electrophoresis under reducing and non-reducing conditions. The purified products were collected and concentrated to nearly 2 mg/ml using 50 kDa cutoff Amicon Ultra-15 centrifugal filter units (Millipore, Cat#UFC905024).

Monoclonal antibodies were purified using affinity chromatography on protein A resin (Cytiva, Cat#17040201) according to the manufacturer’s protocol. Briefly, clarified culture supernatant was purified using 1 ml HiTrap Protein A HP on an ÄKTA Pure chromatographer (Cytiva). The column was washed with 10 column volumes of phosphate-buffered saline (PBS, pH 7.4), and then eluted with 10 column volumes of 0.2 M glycine at pH 3.0. The elution fractions were harvested and immediately neutralized with one fifth of the volume of 1 M HEPES pH 8.0, followed by concentration using 10 kDa cutoff Amicon Ultra-15 centrifugal filter units (Millipore, Cat#UFC901024). The antibodies were finally purified by size exclusion chromatography on a Superdex 200 Increase 10/300 GL column pre-equilibrated with PBS, aliquoted and stored at 4 °C until for use.

The monomeric ACE2, trimeric I53-50A1 and pentameric I53-50B.4PT1 proteins were expressed and purified as previously reported^31,55^. In brief, endotoxin of purifified I53-50A1trimer and I53-50B.4PT1 pentamer was removed by ToxinEraser™ Endotoxin Removal Kit (Genscript, Cat#L00338) following the manufacturer’s instructions prior to assembly. Before formulation in MF59-like adjuvant, residual endotoxin of HexaPro-based immunogens was again tesed using by ToxinSensorTM Chromogenic LAL Endotoxin Assay Kit (Genscript, Cat#L00350). Measured endotoxin concentrations of purifified HexaPro-based immunogens were below 0.1 EU/mL.

### In vitro assembly and purification of mosaic HexaPro-I53-50 nanoparticles

Protein concentrations were determined using bicinchoninic acid (BCA) assay kit (ThermoFisher Scientific, Cat#23225). To assemble the HexaPro-I53-50 nanoparticle with immunogens derived from SARS-CoV-2 prototype and variants, all individual HexaPro-I53-50A-bearing trimeric components were added to an Eppendorf tube and diluted to a concentration of 1 mg/ml with assembly buffer (50 mM HEPES pH 8.0, 300 mM NaCl, 5% glycerol). Then, the components were mixed with an equimolar ratio of I53-50B.4PT1 pentameric components in 50 mM HEPES pH 8.0, 300 mM NaCl, 0.5% (w/v) CHAPS and 5% glycerol at ambient temperature for 1 hour with gentle rocking. After assembly and incubation in vitro, the assembled samples were centrifuged at 17,000×*g* and 4 °C for 10 min to remove aggregated proteins, and then further purified to remove residual unassembled I53-50B.4PT1 by gel filtration chromatography on a Superose 6 Increase 10/300 GL column (Cytiva, Cat#29091596) pre-equilibrated with PBS. The elution fractions containing assembled individual HexaPro-I53-50 nanoparticle immunogens (between 8 and 9.5 ml of column volume) were harvested and pooled, and then concentrated using 100 kDa cutoff Amicon Ultra-15 centrifugal filter units (Millipore, Cat#UFC910024), followed by sterilization and centrifugation at 3900×*g* and 4 °C for 10 min using a 0.22-µm pore size centrifugal filter (Corning, Cat#8160).

To obtain the quadrivalent mosaic SARS-CoV-2 HexaPro-I53-50 nanoparticle immunogens, all four HexaPro-I53-50A-bearing trimeric components were mixed in equimolar ratios at a concentration of 2 mg/ml in assembly buffer (50 mM HEPES pH 8.0, 300 mM NaCl, 5% glycerol) by pipetting, and then added to equimolar I53-50B.4PT1 pentameric components. At 1 hours post incubation at room temperature with gentle rocking, the assembled mosaic HexaPro-I53-50 immunogens were purified the same way as individual HexaPro-I53-50 nanoparticle immunogens. All purified immunogens were sterilized using centrifuge tube filter with 0.22 µm pore size and stored at 4 °C.

### Particle size measurement by dynamic light scattering

Dynamic light scattering analysis was conducted to measure the hydrodynamic diameters of purified protein complexes at 25 °C using a Zetasizer Ultra instrument (Malvern PANalytical) following the manufacturer’s specifications. Briefly, samples were diluted to a final concentration of 0.5 mg/ml in buffer A and applied to 40 µl disposable solvent-resistant micro-cuvette (Malvern PANalytical, Cat#ZEN0040) and left to stand at 25 °C for 2 min. Each sample was measured in quintuplicate. The polydispersity index and hydrodynamic diameter of purified proteins were obtained in Zetasizer Ultra ZS Xplorer version 2.0 and visualized in GraphPad Prism version 8.0.

### Stability analysis by differential scanning fluorescence

Differential scanning fluorescence (DSF) analysis was used to characterize the thermal stability of purified proteins on the UNcle (UNchained Labs) according to the manufacturer’s suggestions. Briefly, the prototypic SARS-CoV-2 HexaPro trimer, four HexaPro-I53-50 nanoparticle immunogens derived from the SARS-CoV-2 prototype and variants, as well as the quadrivalent mosaic HexaPro-I53-50 nanoparticle immunogens were diluted to 0.5 mg/ml in the storage buffer (50 mM HEPES pH 8.0, 300 mM NaCl, and 5% glycerol). Subsequently, 9 µL of each sample was loaded into a quartz capillary cassette (UNi, UNchained Labs) in triplicate. Each sample was measured with four acquisitions, for 5 s per acquisition. The temperature was increased with a thermal ramp from 25 to 95 °C at a rate of 0.6 °C /min. The melting temperature (Tm) and aggregation temperature onset (Tagg) were analyzed using UNcle analysis software version 4.0.

### Negative-staining electron microscopy

HexaPro-based immunogens, including HexaPro-I53-50 nanoparticles of SARS-CoV-2 prototype and variants, as well as mosaic HexaPro-I53-50 nanoparticles, were diluted to 0.25 mg/ml using the storage buffer (50 mM HEPES pH 8.0, 300 mM NaCl and 5% glycerol), and individual I53-50 nanoparticles were diluted to 0.1 mg/ml in PBS. Then, samples comprising 3 µl of the purified protein were added to glow discharged carbon-coated 300 mesh copper grids, incubated for 1 minute at room temperature, and then dipped into PBS. After 1 minutes, excess fluid was blotted away with filter paper. The grids were stained with 2% uranyl acetate, incubated for 45 s, and then air-dried. The grids were inspected and images recorded using a Talos L120C G2 electron microscope (FEI) equipped with a Ceta 16 M 4 K x 4 K CMOS camera.

### Immunoprecipitation

Samples comprising 5 μg of SARS-CoV-2 purified RBD-specific monoclonal antibody S2-E12 at a concentration of 1 mg/ml were incubated with 20 μl of protein A beads at room temperature for 30 minutes. After washing thrice with PBS, 5 μg HexaPro, individual HexaPro-I53-50A1 and HexaPro-I53-50 nanoparticle immunogens of SARS-CoV-2 prototype and three additional variants, cocktail HexaPro-I53-50 as well as mosaic HexaPro-I53-50 were added to the S2-E12 bound beads and incubated for an additional hour in PCR reaction tubes with gentle rocking. The beads were washed thrice with PBS to remove unbound proteins, and then separated from the supernatant by centrifugation at 1000×*g* for 3 min, followed by heating to 100 °C for 5 min in 4× Laemmli sample buffer containing 20 mM dithiothreitol. Beads binding to monoclonal antibodies and immunogen mixtures were analyzed by SDS-PAGE stained with coomassie blue solution.

### Antigenic characterization

Enzyme-linked immunosorbent assay (ELISA) was conducted to evaluate the binding characteristics of HexaPro-based nanoparticle immunogens and SARS-CoV-2 specific monoclonal antibodies (REGN-10933, Regdanvimab, S2-E12, COVA1-16, S2-H14, S2-M11, CB6, IgG1-ab1, P2B-2F6, CR3022, COV2-2196, 4A8, Fab 2-15, and REGN-10987), and AMMO1 as the antibody homotypic control (anti-EBV gH/gL). The 96-well high binding microplates were pre-coated with 1 μg/ml of immunogens (0.1 ml/well) at 4 °C overnight. The plates were rinsed once with PBS containing 0.05% tween 20 (PBST) using a plate washer (Tuopu), and blocked with PBS containing 5% casein and 2% gelatin. Then, monoclonal antibodies in a tenfold serial dilution in PBS at an initial concentration of 10 μg/ml and a final concentration of 0.000001 μg/ml were added to the wells in duplicate, and incubated for 2 h at 37 °C. The assay plates were washed five times with PBST, after which a horseradish peroxidase (HRP)-conjugated goat anti-human IgG secondary antibody (Southern Biotech, Cat#2015-05) at a 1:5000 dilution was added to the wells and incubated for 45 min at 37 °C. The wells were developed by the addition of soluble 3,3′,5,5′-tetramethylbenzidine substrate (TMB, Tiangen, Cat#PA107-01) for 15 min, after which the colorimetric reaction was stopped by adding 1:12 diluted concentrated hydrochloric acid. The absorbance of the plates at 450 and 630 nm was measured using an Epoch^TM^ 2 microplate spectrophotometer (BioTek). EC_50_ values were calculated from plotted and fitted sigmoid curves using Prism v.8.0 software (GraphPad) after monoclonal antibody concentrations were converted to logarithms using four-parameter nonlinear regression.

### Biolayer interferometry

To determine the antigenicity of HexaPro protein variants and HexaPro-based nanoparticles or to determine the capacity of serum competition with ACE2 in a quantitative manner, we performed BLI on an Octet R8 instrument (Sartorius) following the manufacturer’s protocol. For antigenicity determination, we performed standard kinetic assays to calculate the K_D_ (equilibrium dissociation constant) of the indicated HexaPro or HexaPro-based nanoparticles binding to antibody or ACE2. Briefly, antibody or ACE2 was firstly immobilized onto the streptavidin biosensors (Sartorius, Cat#18-5021). Then, serially diluted HexaPro or HexaPro-based nanoparticle was bound to the biosensors for 180 seconds, followed by 300 seconds of equilibration with kinetic buffer (PBS with 0.1% Tween 20). Protein A biosensors could be reused after a regeneration step with 0.2 M glycine pH 3.0 as washing buffer after each kinetic assay. The binding curves were analyzed and fitted with a 1:1 model in Octet Analysis Studio (Sartorius) for calculation of kinetic parameters.

For serum competition determination, a manual competitive assay was performed as follows. Serum samples from non-human primates after the 2^nd^ booster immunization were retrieved and samples from each non-human primate (5 µL each) in the same group (WT NP or Mosaic NP) were mixed in representation of the overall group. Additionally, serum samples from all 8 non-human primates before primary immunization were mixed and used as the background serum control. Then, the same amount of each HexaPro of variants was immobilized onto the biosensors, after which PBS or 1:1000 diluted serum samples from the background serum control group, WT NP group or Mosaic NP group were loaded for 200 seconds to occupy the spike proteins on the biosensors. Then, 500 nM ACE2 proteins were associated with the biosensors for another 200 seconds. If the serum could predominantly occupy target spike proteins, the secondary binding of ACE2 would be low or even absent, indicating a protective effect of serum by blocking the in vitro interaction between spike protein and ACE2. The raw curves were aligned to the start point of the ACE2 binding phase and retrieved using Octet Analysis Studio.

### Generation of pseudotyped viruses

A total of 26 human immunodeficiency virus-based pseudotyped viruses, including SARS-CoV-2 prototype, 5 VOCs (B.1.1.7, B.1.351, P.1, B.1.617.2, and B.1.1.529), 1 VOIs (C.37), 1 VUMs (B.1.525), single or combinatorial key residue mutations in the RBD and 6 coronavirus-related strains (SARS-CoV, HCoV-229E, MERS-CoV, HCoV-NL63, HCoV-OC43, and RATG13), were used to evaluate the neutralizing activity of sera. Pseudotyped virions were packaged as previously described^46,56^, with minor modifications as follows. Briefly, HEK293T cells were seeded in 15-cm dishes and cultured overnight to achieve 70-80% confluency. The following day, the cells were co-transfected with 10 μg expression plasmids of the Env-defective, luciferase-encoding HIV-1 genome (pNL-4.3-Luc-R-E-) and 10 μg plasmids encoding wild-type or mutated SARS-CoV-2 spike protein using PEI-MAX (Polysciences, Cat#24765-1). Six hours post-transfection, the cell supernatant was replaced with fresh complete DMEM medium. Following another 48 h, the supernatant was harvested, clarified by centrifugation at 200×*g* to remove cell debris, and stored at −80 °C before use. SARS-CoV, HCoV-229E, MERS-CoV, HCoV-NL63, HCoV-OC43 and BCoV-RATG13 pseudotyped viruses were also generated the same as above to examine the cross-reaction of neutralizing antibodies from the sera of vaccinated non-human primates.

To titrate the pseudotyped virus stocks, 100 µl virus stock samples were serially diluted twofold with DMEM medium supplemented with 2% FBS and 2 µg/ml puromycin in duplicate, incubated 48 h with hACE2-HEK293T cells in 96-well Nunclon Delta-treated flat bottom microporous plates (ThermoFisher Scientific, Cat#167008), after which the medium was gently aspirated. Cells were lysed with 50 µl 1× lysis buffer containing Steady-Glo luciferase substrate (Promega, Cat#E2520), and incubated at room temperature for 10 min, with shaking at 40 rpm. Samples comprising 45 µl of the cell lysates were transferred to white plates (Corning, Cat#3917), and luciferase activity was measured as relative luciferase units (RLU) using a GloMax Navigator GM2010 luminometer (Promega) according to the manufacturer’s instructions.

### Immunizations and challenge of mice with SARS-CoV-2 prototype or the B.1.351 variant

A total of 120 6-week-old specific pathogen-free (SPF) male BALB/c mice were purchased from Zhejiang Vital River Laboratory Animal Technology Co., Ltd, and raised in the laboratory animal facilities at Sun Yat-sen University Cancer Center. Mice were housed under standard humidity (55 ± 10%) and temperature (21 ± 1 °C) conditions in a 12-hour dark/light cycle with enough food and water. All animal studies were performed in accordance with recommendations for care and use of laboratory animals, and were approved by the Institutional Animal Care and Use Committees of the Guangzhou Medical University and of the Sun Yat-sen University Cancer Center (approval number: L102042020000A). Mice were randomly divided into 6 groups (20 per dosage group) 1 week prior to immunization. The night before immunization, immunogens in PBS were mixed with an equal volume of MF59-like adjuvant and incubated overnight at 4°C with gentle rocking. Mice were immunized subcutaneously on the abdominal skin with 5 µg SARS-CoV-2 prototype HexaPro or 6.5 µg (equimolar amount to 5 µg SARS-CoV-2 HexaPro) SARS-CoV-2 prototype HexaPro-I53-50, cocktail HexaPro-I53-50 and mosaic HexaPro-I53-50 nanoparticle immunogens at weeks 0 and 3. Two weeks after primary and booster immunizations, the mice were bled, sera were harvested, placed into a 37 °C incubator for 30 min, centrifuged at 17,000×*g* for 10 min to remove the red blood cells, and subjected to heating at 56 °C for 30 min to inactivate pathogens and complement proteins. Serum samples were stored at −80 °C for immunological and virological assays. To investigate the protection efficacy against SARS-CoV-2 prototype and B.1.351 strain infection exerted by immunization, mice from each group as stated above were again randomly divided into two groups (10 per dosage group) 3 weeks after the booster immunization. Immunized mice (aged 12 weeks old) challenged with SARS-CoV-2 authentic virus were anesthetized with isoflurane and transduced intranasally with 75 µl DMEM containing 2.5×10^8^ FFU of Ad5-hACE2. Five days later, all mice were exported to the BSL3 facility at the Guangzhou Customs District Technology Center for challenge assays, anesthetized with isoflurane, and then inoculated intranasally with 50 µl 2×10^6^ plaque-forming units (PFU)/ml SARS-CoV-2 prototype and 2 × 10^6^ PFU/ml SARS-CoV-2 B.1.351 variant authentic virus. After infection, the body weight of each mouse was recorded daily for 8 days. Two days later, 4 mice in each group were euthanized and the lungs were harvested to examine the virus titers. Four days post challenge, the mice were anesthetized, transcardially perfused with PBS, and fixed with 25 ml fresh zinc formalin for 7 days at ambient temperature for subsequent histological and immunohistochemical examinations.

### Immunization of cynomolgus macaques

A total of 8 14-month-old cynomolgus macaques (*Macaca fascicularis*, 6 females and 2 males, body weight ranging from 1.5 to 1.8 kilograms) were purchased from Zhanjiang Prima Biotech Inc., and divided into two groups (4 per group), followed by maintenance at the Primate Research Center of the Institute of Zoology, Guangdong Academy of Sciences in compliance with standard procedures and regulations of the Committee on the Care and Use of Laboratory Animals. The immunization protocol was reviewed and approved by the Institutional Animal Care and Use Committees of the Institute of Zoology, Guangdong Academy of Sciences (approval number: GZZ20201201). All cynomolgus macaques were tested negative for Simian Retrovirus D, Simian Immunodeficiency Virus, Simian T Lymphotropic Virus Type 1 and Monkey B virus by ELISA. The cynomolgus macaques were immunized intramuscularly with 65 µg of WT NP or Mosaic NP immunogens (equimolar amount to 50 µg of SARS-CoV-2 HexaPro) in the presence of MF59-like adjuvant at weeks 0, 4, and 8. A total volume of 1 ml adjuvanted vaccine was injected into the hind leg at multiple sites for each animal. Serum samples were collected at 2-week intervals until 10 weeks, and heat-inactivation at 56 °C for 30 min, followed by storage at −80 °C for immunological and virological assays.

### Serum ELISA binding assays

To test the binding abilities of antibodies from the sera of immunized mice and non-human primates, a panel of 6 purified recombinant HexaPro prototype and variants, including SARS-CoV-2 wild type, Alpha, Beta, Gamma, Delta and Eta, were used as antigens. Briefly, recombinant antigen in PBS was coated onto the 96-well microplates (30 ng/well) and incubated overnight at 4 °C. The next day, the plates were washed and blocked as stated above. Heat-inactivated serum samples from immunized mice (starting at 1:100 dilution) or non-human primates (starting at 1:10 dilution) were ten- or fivefold diluted with PBS and added to the plates, respectively. After incubation for a period of 1 hour at 37 °C, the plates were rinsed five times with PBST, and then incubated with a 1:5000 dilution of horseradish peroxidase (HRP)-conjugated goat anti-mice (Abcam, Cat#ab6789) or anti-human (Southern Biotech, Cat#2015-05) IgG secondary antibody in PBS containing 5% casein and 2% gelatin for 45 min at 37 °C. After rinsing five times with PBST using a plate washer (Tuopu), the plates were developed with TMB substrate for10 min at 37 °C, and the reaction was stopped with 1 M hydrochloric acid. The absorbance at a wavelength of 450 nm (*A*_450_) and 630 nm (*A*_630_) was recorded, and the difference between the *A*_450_ and *A*_630_ was calculated using an Epoch^TM^ 2 microplate spectrophotometer (BioTek). ELISA binding endpoint titers were defined as the highest reciprocal of the serum dilution at which the absorbance exceeded a cutoff of 0.2.

### Pseudovirus neutralization assay

For the pseudotyped virus-based neutralization assay, immunized mice sera at a starting dilution of 1:40 (eight dilutions) and sera collected from non-human primates at a starting dilution of 1:10 (eight dilutions) were fourfold diluted with DMEM medium supplemented with 2% FBS and 2 µg/ml puromycin, and then mixed with an equal volume of pre-titrated pseudotyped virus. After incubation at 37 °C for 2 h, the mixtures of sera and pseudotyped virus were added to the 96-well Nunclon Delta-treated flat bottom microporous plates pre-seeded with 1.5 × 10^4^ hACE2-HEK293T cells the night before. The plates included 16 wells with uninfected cells without virus addition and cells infected with pseudotyped virus without antibodies, as negative and positive control, respectively. After incubation for an additional 48 h, the cells were washed once with PBS and lysed, after which the firefly luciferase signals of infected cells were measured as detailed above. Percent inhibition was determined by subtracting the average uninfected cell luminescence (negative control) and dividing by the average infected cell luminescence in the presence of pseudotyped virus (positive control). The 50% inhibitory concentration (IC_50_) for each sample was calculated from fitted sigmoidal curves with 4-parameter nonlinear regression using GraphPad Prism v.8.0 software.

### Authentic SARS-CoV-2 virus neutralization assay

A total of 6 SARS-CoV-2 live viruses, including prototype, VOCs (Alpha, Beta, Gamma and Delta) and a VUM (Eta), were used to evaluate the neutralization activity of sera at a BSL-3 facility in Guangdong Provincial Center for Disease Control and Prevention. Briefly, heat-inactivated serum samples from immunized mice (starting at 1:40 dilutions, six dilutions) or serum samples from immunized non-human primates (starting at 1:16 dilutions, six dilutions) were fourfold diluted with completed DMEM medium, mixed with an equal volume of 100 median tissue culture infective dose (TCID_50_), and then incubated at 37 °C for 2 h. Mock infected cells (cells only) and live virus-infected cells (virus and cells without serum) were included negative and positive control, respectively. Subsequently, 100 µl mixtures containing serum samples and virus were transferred and added to the pre-seeded Vero-E6 cells with a density of 2.0 × 10^4^ cells per well in 96-well plates (Corning, Cat#3599). The plates were incubated at 37 °C in a CO_2_ incubator for an additional 3 to 5 days, after which the cytopathic effect (CPE) induced by the virus was observed under an optical microscope at 40-fold magnification. The neutralizing titer of the serum sample against SARS-CoV-2 authentic virus was defined as the highest reciprocal value of the serum dilution required for 50% inhibition of viral infection using the Reed–Muench method^57^.

### Focus forming assay from BALB/c mice lung homogenates

Virus titers in lung tissues post challenge were tested using the FFA as previously described^58^. Briefly, tissues were weighted, homogenized and tenfold diluted with DMEM starting from 1:3 dilution, and then added to wells pre-seeded with Vero-E6 cells in 96-well plates. After 1 h post inoculation, the cell supernatant containing the inoculum was removed and 125 μl of 1.6% carboxymethylcellulose was added to the well. After 24 hours of incubation, 4% paraformaldehyde was added to the cell monolayer for fixation at ambient temperature for 2 h, followed by permeabilization with 0.2% Triton X-100. Subsequently, the plates were washed once with PBS and incubated for 1 h with a rabbit anti-SARS-CoV-2 nucleocapsid primary antibody (Sino Biological, Inc., Cat#40143-T62) at a 1:12000 dilution, followed by HRP-conjugated goat anti-rabbit secondary antibody (Jackson ImmunoResearch Laboratories, Inc., Cat#111-035-144) at a 1:6000 dilution. The foci in each well were developed with TrueBlue Peroxidase substrate (KPL, Cat#510-0030), and counted automatically using an ELISPOT reader (Cellular Technology Ltd.). The viral titer of the lung tissue was expressed as FFU per gram tissue.

### Measurement of viral burden

To measure the SARS-CoV-2 copy number in infected mice, the lungs were harvested, weighted and homogenized for RNA extraction using TRIZOL regent (Sigma-Aldrich, Cat#T924) following the manufacturer’s instructions. Complementary DNA was then synthesized via reverse transcription with 1 µg extracted total RNA as template in a 20 µl reaction mixture containing GoScript™ Reverse Transcriptase (Promega, Cat#A5001). The SARS-CoV-2 copy number was then quantified using a TaqMan real-time PCR assay with the iTaq Universal Probe SuperMix (Bio-Rad, Cat#1725131) and the following primers and probes: Target 1 (open reading frame 1ab, *ORF1ab*): Forward primer: 5′-CCCTGTGGGTTTTACACTTAA-3′; Reverse primer: 5′-ACGATTGTGCATCAGCTGA-3′; Probe: 5′-FAM-CCGTCTGCGGTATGTGGAAAGGTTATGG-BHQ1-3′; Target 2 (nucleoprotein, *N*): Forward primer: 5′-GGGGAACTTCTCCTGCTAGAAT-3′; Reverse primer: 5′-CAGACATTTTGCTCTCAAGCTG-3′; Probe: 5′-FAM-TTGCTGCTGCTTGACAGATT-TAMRA-3′. The 2^-ΔΔCq^ method was used to calculate the relative RNA levels, using the housekeeping gene β-actin for normalization. Mice that were administered PBS instead of being challenged with SARS-CoV-2 were included as a negative control, and the tenfold serial dilution of the synthetic plasmid template (PUC57-ORF1ab-N plasmid) with a known concentration was measured together to generate a standard curve for the calculation of absolute copy numbers.

### Histopathology and immunohistochemistry

Lung tissues were fixed with freshly prepared zinc formalin for 7 days, moved to 75% ethanol for dehydration, embedded in paraffin at the melting point of 55 °C, and cut to 5-µm sections per sample. The sections were baked at 60 °C for 2 h, and de-waxed in xylene for 10 min and then rehydrated by placing into a decreasing concentration series of graded ethanol solutions and distilled water, followed by staining with hematoxylin and eosin. For immunohistochemical analysis, lung sections were heat-treated for epitope retrieval in a pressure cooker with citrate buffer (pH 6.0), and incubated with freshly prepared 3% hydrogen peroxide for 30 minutes to block endogenous peroxidases, followed by incubation with blocking buffer. The slides were incubated with a rabbit anti-SARS-CoV-nucleoprotein polyclonal antibody (Novus, Cat#NB100–56576) at 1:1000 dilution overnight at 4 °C, and then incubated with a 1:2000 dilution of HRP-conjugated goat anti-rabbit IgG (Abcam, Cat#ab205718) at ambient temperature for 45 min. Subsequently, diaminobenzidine was added to the lung sections, followed by counterstaining with hematoxylin, and then rinsed in distilled water. The microscopic images were captured using a Nikon Eclipse Ni-U optical microscope.

### Measurement of cytokine and chemokine mRNAs in mouse lungs

Following two days after challenge, the mice were euthanized and lungs were collected for homogenization. Total RNA was isolated from the homogenized lungs using Trizol reagent (Sigma-Aldrich, Cat#T924), following the manufacturer’s instructions, and was then used as the template for reverse transcription using a kit as described above (Promega, Cat#A501), with 1 µg RNA in a 20 µl reaction mixture. The relative expression levels of different cytokines and chemokines in the lungs were measured by real-time quantitative PCR using the LightCycler 480 SYBR Green I MasterMix (Roche, Cat#4887352001). Primers for the detection of the indicated genes were synthesized by RuiBiotech and listed in Supplementary Table 2.

### Quantification and statistical analysis

All statistical analysis were conducted by using Prism v.8.0 software (GraphPad). Statistical significance of each group in binding and neutralizing antibodies was compared and analyzed using the two-tailed Mann–Whitney *U* test. In the mice challenge studies, Kruskal–Wallis ANOVA with Dunn’s correction was used to evaluate the statistical significance in inflammatory cytokine expression and viral copies across vaccinated HexaPro immunogens groups compared to the PBS-treated control group. Significant differences for each comparison were indicated in each figure legend. A *p* value < 0.05 was statistically significant (*p < 0.05, **p < 0.01, ***p < 0.001).

### Reporting summary

Further information on research design is available in the Nature Research Reporting Summary linked to this article.

## Supplementary information

Supplementary Information

Reporting Summary

## Data Availability

All data used in this study are available in this paper and supplementary information, or available from the corresponding authors under reasonable request. The accession numbers of spike of coronavirus referred in this work are obtained from Genbank or GISAID database. The key experimental data was deposited into Research Data Deposit (https://www.researchdata.org.cn) with serial number RDDB2022613701. Source data are provided with this paper.

## Source data

Source Data
