# Supplementary material for: Quadrivalent mosaic HexaPro-bearing nanoparticle vaccine protects against infection of SARS-CoV-2 variants
Source: Nat Commun. 2022 May 13;13:2674. doi: 10.1038/s41467-022-30222-w (PMC9106700; doi:10.1038/s41467-022-30222-w)
Supplement: Supplementary file 1 — Supplementary Information [file 41467_2022_30222_MOESM1_ESM.pdf]

Supplementary Information for

Yin-Feng Kang[1#], Cong Sun[1#], Jing Sun[2#], Chu Xie[1], Zhen Zhuang[2], Hui-Qin Xu[3], Zheng Liu[3], Yi-Hao Liu[4,5], Sui Peng[4], Run-Yu Yuan[6✉], Jin-Cun Zhao[2,7✉], Mu-Sheng Zeng[1,8✉]

[1] State Key Laboratory of Oncology in South China, Collaborative Innovation Center for Cancer Medicine, Guangdong Key Laboratory of Nasopharyngeal Carcinoma Diagnosis and Therapy, Department of Experimental Research, Sun Yat-sen University Cancer Center (SYSUCC), Sun Yat-sen University, Guangzhou, 510060, P. R. China.

[2] State Key Laboratory of Respiratory Disease, National Clinical Research Center for Respiratory Disease, Guangzhou Institute of Respiratory Health, The First Affiliated Hospital of Guangzhou Medical University, Guangzhou, 510182, P. R. China.

[3] Cryo-electron Microscopy Center, Southern University of Science and Technology, Shenzhen, 518000, P. R. China.

[4] Institute of Precision Medicine, Clinical Trials Unit, The First Affiliated Hospital of Sun Yat-sen University, Guangzhou, 510080, P. R. China.

[5] Department of Endocrinology, The First Affiliated Hospital of Sun Yat-sen University, Guangzhou, 510080, P. R. China.

[6] Guangdong Provincial Institution of Public Health, Guangdong Provincial Center for Disease Control and Prevention, Guangzhou, 511430, P. R. China.

[7] Guangzhou Laboratory, Bio-island, Guangzhou, 510320, P. R. China.

[8] Guangdong-Hong Kong Joint Laboratory for RNA Medicine, Guangzhou, 510120, P. R. China.

[#] These authors contributed equally: Yin-Feng Kang, Cong Sun, Jing Sun.

[✉] email: cecilia_yry@hotmail.com; zhaojincun@gird.cn; zengmsh@sysucc.org.cn

Supplementary Figures

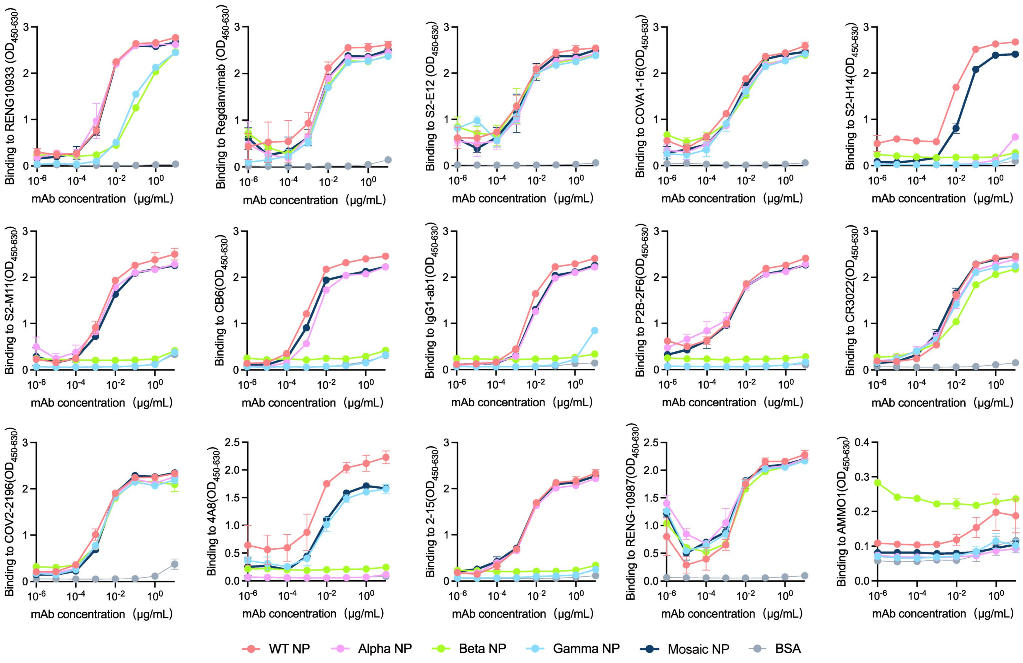

Supplementary Fig. 1. Binding curves of SARS-CoV-2 Spike-specific neutralization antibodies against HexaPro-based nanoparticle immunogens measured by ELISA. The data are presented as means ± SD in duplicate from three independent experiments SARS-CoV spike-specific CR3022 antibody and EBV gH/gL-specific AMMO1 antibody were used as controls. Source data are provided as a Source Data file.

a

	Tm_1 (° C)	Tm_2 (° C)	Tm_3 (° C)	Tm_4 (° C)	T_{agg} 266 (° C)
WT HexaPro	33.9	57.9	70.1	-	-
WT NP	36.4	64.6	76.1	-	-
Alpha NP	34.7	65.8	74.5	-	-
Beta NP	33.9	65.2	74.9	-	-
Gamma NP	36.6	46.4	63.0	74.5	44.3
Mosaic NP	35.1	44.8	65.7	76.8	51.3

b

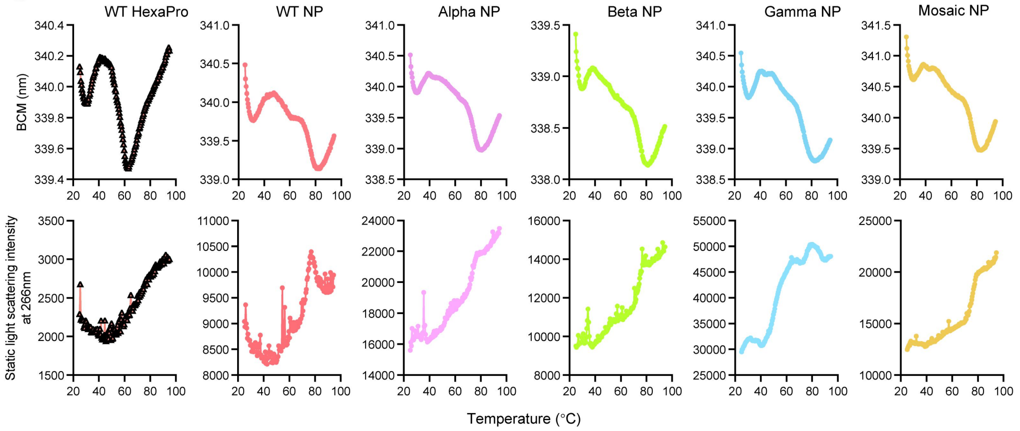

Supplementary Fig. 2. Thermostability analysis of HexaPro-based immunogens.

a. Thermostability parameter table of SARS-CoV-2 HexaPro or Hexapro-based nanoparticles determined by DSF from three replicate experiments. Tm: melting temperature; Tagg 266: aggregation temperature identified by the static light scattering at 266nm.

b. Barycentric mean (BCM) of the intrinsic protein fluorescence from 300-430nm and static light scattering intensity at 266nm of the HexaPro-based immunogens. The curves presented the lined mean data of each temperature point from three replicate experiments. Source data are provided as a Source Data file.

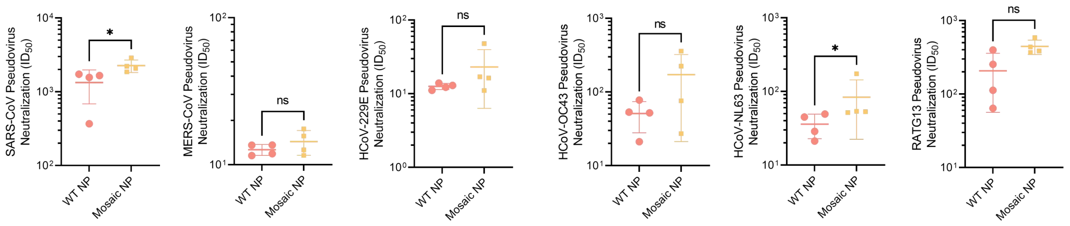

Supplementary Fig. 3. Cross-neutralization of bat and human coronaviruses by sera elicited by HexaPro-based nanoparticle immunogens. Sera were collected from two weeks after the second booster dose (n=4 cynomolgus macaques in each group) and used to measure the cross-neutralization antibody titers using the pseudoviruses assay. The data were expressed as means ±SD. Comparison between the two groups were performed using a two-tailed Mann-Whitney U test. Mosaic NP vs WT NP in SARS-CoV pseudovirus neutralization titers *p = 0.0286, Mosaic NP vs WT NP in HCoV-NL63 pseudovirus neutralization titers *p = 0.0286. *p < 0.05; ns, no significant. Source data are provided as a Source Data file.

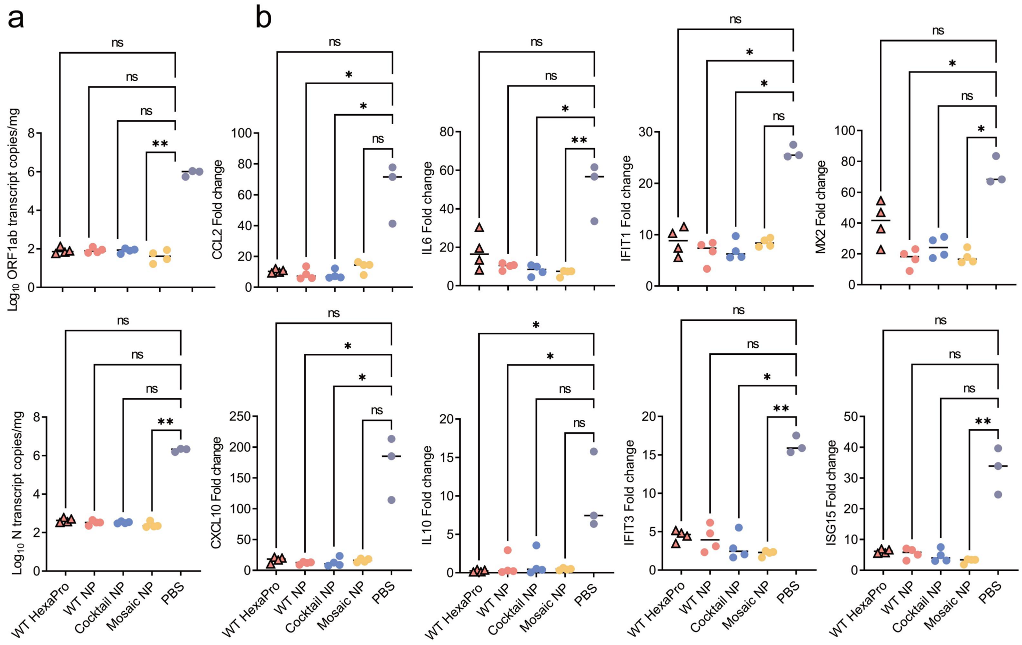

Supplementary Fig. 4. Expression of immune-related cytokines and chemokines and viral burden in infected lung tissues at 2 days post B.1.351 variant strain infection. a and b. The expression levels of viral burden (a) and cytokines and chemokines (b) in the lungs at 2 days post infection was measured by qRT-PCR. The data were expressed as means ± SD. Comparisons between the two groups were performed using a Kruskal-Wallis ANOVA with Dunn's correction. *p < 0.05, **p < 0.01; ns, no significant. (a) Mosaic NP vs PBS in *ORF1ab* and *N* transcript copies, **p = 0.0082. (b) WT NP and Cocktail NP vs PBS in *CCL2* fold change, *p = 0.0175, *p = 0.0210, respectively. Cocktail NP and Mosaic NP vs PBS in *IL6* fold change, *p = 0.0250, **p = 0.0055, respectively. WT NP and Cocktail NP vs PBS in *IFIT1* fold change, *p = 0.0354, *p = 0.0298, respectively. WT NP and Mosaic NP vs PBS in *MX2* fold change, *p = 0.0175, *p = 0.0145, respectively. WT NP and Cocktail NP vs PBS in *CXCL10* fold change, *p = 0.0250. WT HexaPro and WT NP vs PBS in *IL10* fold change, *p = 0.0298, *p = 0.0419, respectively. Cocktail NP and Mosaic NP vs PBS in *IFIT3* fold change, *p = 0.0419, **p = 0.0067, respectively. Mosaic NP vs PBS in *ISG15* fold change, **p = 0.0082. Source data are provided as a Source Data

file.

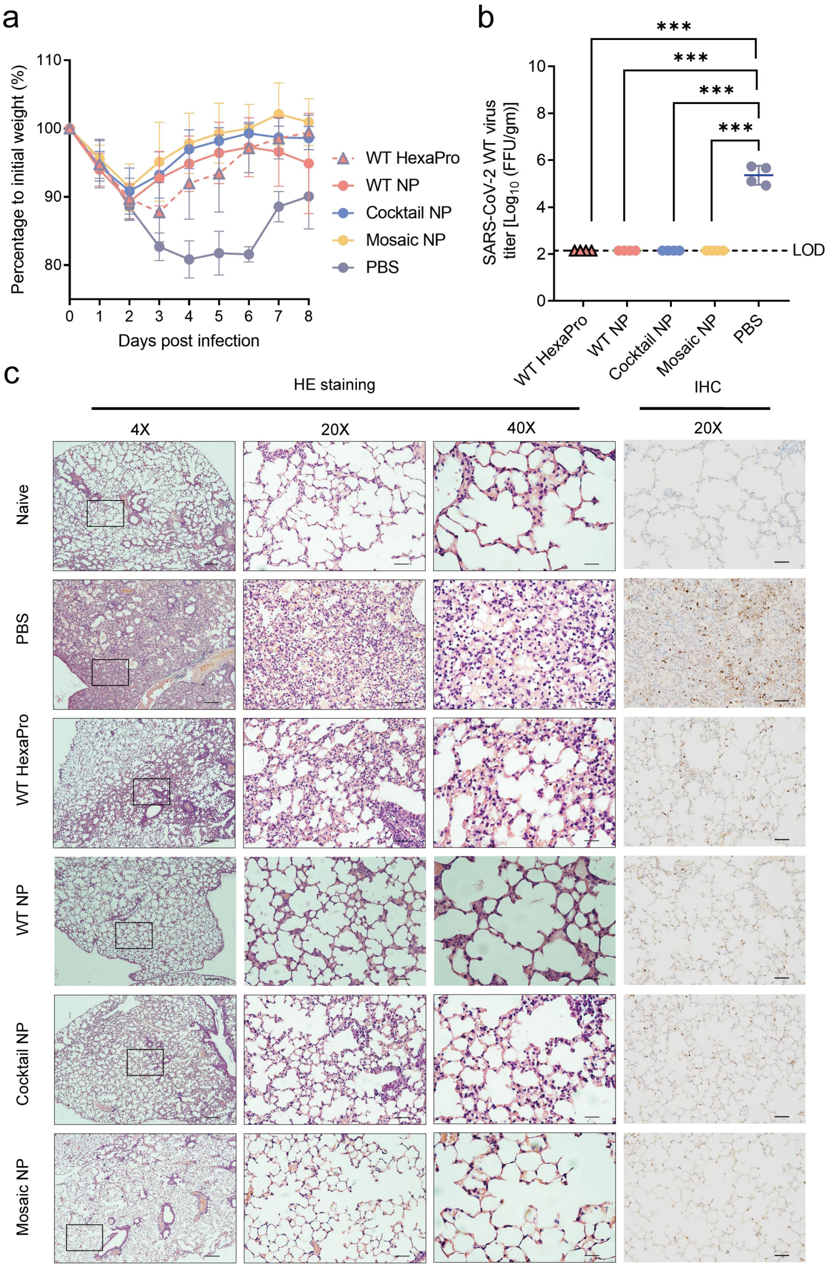

Supplementary Fig. 5. Protective efficacy of HexaPro-bearing immunogens in mice following challenge with authentic ancestral SARS-CoV-2 virus *in vivo*. a-c, six-week-old male BALB/c mice were subcutaneously immunized with an equivalent amounts of HexaPro-based immunogens (equal to 5 µg HexaPro) at weeks 0 and 3. At 3 weeks after the second vaccination, the mice were transduced intranasally with 2.5×10^{8} FFU of Ad5-hACE2, and after 5 days of transduction, the mice were intranasally inoculated with 2×10^{6} PFU/ml of authentic ancestral SARS-CoV-2 virus.

Lung tissues were collected for virus titer quantification and clinicopathological analysis.

a. Body weight change of mice (n=3 in PBS-treated and WT NP-vaccinated mice, n=4 mice in WT HexaPro, Cocktail NP and Mosaic NP-vaccinated group) after infection with ancestral SARS-CoV-2. The body weight of each mouse was recorded daily for 8 days.

b. Virus titers of lung tissues from mice challenged with ancestral SARS-CoV-2 (n= 4 mice in each group) at 2 days post challenge. LOD, limit of detection. Statistical significance was determined by two-tailed unpaired t test. WT HexaPro, WT NP, Cocktail NP and Mosaic NP vs PBS, ***p = 0.0001.

c. Immunohistological analysis of lung tissues from mice challenged with ancestral SARS-CoV-2 at 4 days post challenge (n=2 mice in each experimental group). Hematoxylin and eosin staining (HE, left) and immunohistochemistry (IHC, right) microscopic images are shown in the figure at magnification. Scale bar for H&E, 250 μm (left); 50 μm (middle); 25 μm (right), Scale bar for IHC, 50 μm. In a and b, the data were expressed and plotted as means ± SD. Source data are provided as a Source Data file.

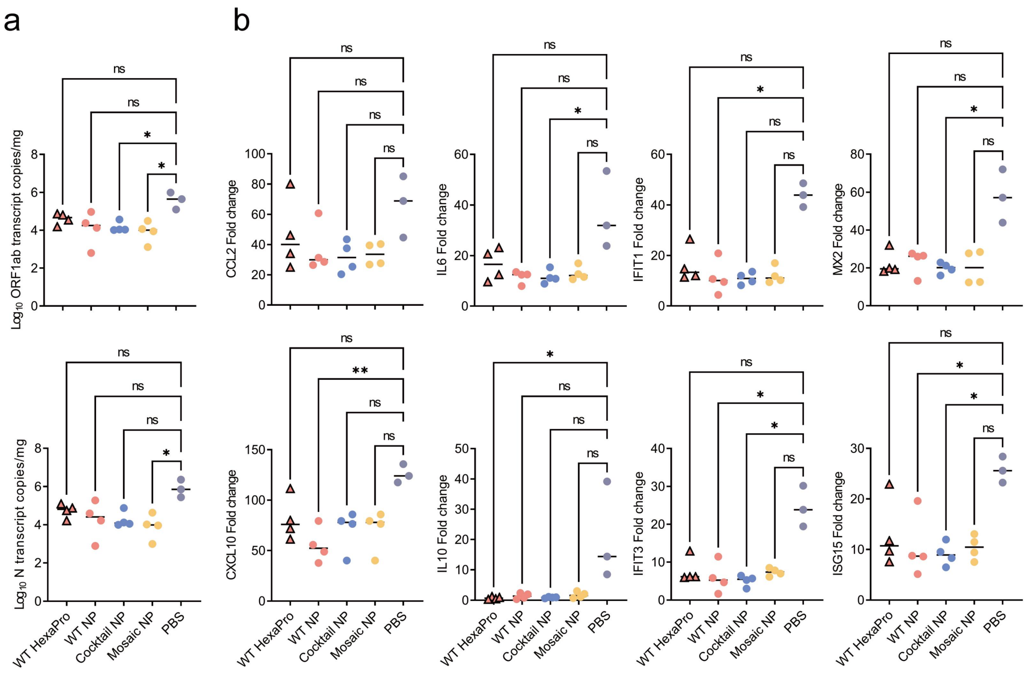

Supplementary Fig. 6. Expression of immune-related cytokines and chemokines and viral burden in infected lung tissues at 2 days post infection with the ancestral SARS-CoV-2 strain. a and b. The expression levels of viral burden (a) and cytokines and chemokines (b) in lungs at 2 days post infection were measured by qRT-PCR assay. The data were expressed as means ± SD. Comparison between the two groups was performed using a Kruskal-Wallis ANOVA with Dunn's correction. *$p < 0.05$; **$p < 0.01$; ns, no significant. (a) Cocktail NP and Mosaic NP vs PBS in *ORF1ab* transcript copies, *$p = 0.0419$ and *$p = 0.0175$, respectively, Mosaic NP vs PBS in *N* transcript copies, *$p = 0.0120$. (b) Cocktail NP vs PBS in *IL6* fold change, *$p = 0.0298$. WT NP vs PBS in *IFIT1* fold change, *$p = 0.0354$. Cocktail NP vs PBS in *MX2* fold change, *$p = 0.0495$. WT NP vs PBS in *CXCL10* fold change, **$p = 0.0098$. WT HexaPro vs PBS in *IL10* fold change, **$p = 0.0210$. WT NP and Cocktail NP vs PBS in *IFIT3* fold change, *$p = 0.0250$, *$p = 0.0145$, respectively. WT NP and Cocktail NP vs PBS in *ISG15* fold change, *$p = 0.0495$. Source data are provided as a Source Data file.

Supplementary Tables

Supplementary Table 1: Kinetic analysis of SARS-CoV-2 mAbs and ACE2 receptor to HexaPro and HexaPro-based nanoparticle immunogens of SARS-CoV-2 prototype and variants.

		Antibody									Receptor		
		REGN10933			P2B-2F6			4A8			ACE2		
		K_D	K_{on}	K_{off}	K_D	K_{on}	K_{off}	K_D	K_{on}	K_{off}	K_D	K_{on}	K_{off}
HexaPro	Wild type	8.691E-11	2.472E05	2.149E-05	3.503E-11	3.419E05	1.198E-05	3.641E-11	3.608E05	1.314E-05	1.104E-08	9.378E04	1.035E-03
	Alpha	<1.0E-12	1.792E05	1.220E-07	<1.0E-12	2.219E05	1.676E-07	3.336E-09	2.339E05	7.802E-04	3.307E-09	1.951E05	6.450E-04
	Beta	6.492E-09	1.523E05	9.890E-04	5.715E-12	4.245E04	2.426E-07	1.583E-09	8.940E04	1.416E-04	4.607E-12	3.306E04	1.523E-07
	Gamma	4.737E-09	2.754E05	1.304E-03	2.760E-09	1.199E05	3.309E-04	<1.0E-12	2.972E05	2.041E-07	2.143E-09	1.178E05	2.524E-04
		K_D	K_{on}	K_{off}	K_D	K_{on}	K_{off}	K_D	K_{on}	K_{off}	K_D	K_{on}	K_{off}
HexaPro-based nanoparticle	Wild type	1.231E-12	1.631E05	2.007E-07	<1.0E-12	1.958E05	1.610E-07	<1.0E-12	2.008E05	<1.0E-07	4.498E-12	3.076E04	1.384E-07
	Alpha	1.106E-12	1.594E05	1.762E-07	1.293E-12	1.878E05	2.429E-07	<1.0E-12	1.596E05	1.528E-07	7.592E-12	3.198E04	2.428E-07
	Beta	4.143E-12	3.399E04	1.408E-07	2.921E-12	3.760E04	1.098E-07	1.276E-12	4.892E04	<1.0E-07	3.181E-12	6.566E04	2.089E-07
	Gamma	<1.0E-12	1.660E05	<1.0E-07	1.493E-12	1.627E05	2.429E-07	<1.0E-12	1.855E05	1.318E-07	1.707E-09	1.012E05	1.728E-04
	Mosaic	1.187E-12	1.750E05	2.077E-07	1.351E-12	1.841E05	2.488E-07	<1.0E-12	1.578E05	<1.0E-07	3.529E-12	5.341E04	1.885E-07

For antibody or receptor binding, SARS-CoV-2 RBD-directed mAbs (REGN10933 and P2B-2F6), NTD-directed mAb (4A8) and ACE2 receptor was immobilized onto the biosensors, and then twofold diluted HexaPro or HexaPro-based nanoparticle immunogens injected into the wells. Binding kinetics were analyzed with a 1:1 model in Octet Analysis Studio. Affinity constant (K_D), kinetic constants of associations (K_{on}) and dissociations (K_{off}) were summarized in this table. K_D is calculated from K_{off} / K_{on}.

Supplementary Table 2: Quantitative RT-PCR primer used in this study.

Gene	Primer	Sequence
CCL2	Forward primer	5'-TTGACCCGTAAATCTGAAGCTAAT-3'
	Reverse primer	5'-TCACAGTCCGAGTCACACTAGTTCAC-3'
IL6	Forward primer	5'-CACTTCACAAGTCGGAGGCT-3'
	Reverse primer	5'-CTGCAAGTGCATCATCGTTGT-3'
IFIT1	Forward primer	5'-CAAGGCAGGTTTCTGAGGAG-3'
	Reverse primer	5'-GACCTGGTCACCATCAGCAT-3'
MX2	Forward primer	5'-ACCAGAGTGCAAGTGAGGAGCT-3'
	Reverse primer	5'-GTACTAGGGCAGTGATGTCCTG-3'
CXCL10	Forward primer	5'-CCTGCCCACGTGTTGAGAT-3'
	Reverse primer	5'-TGATGGTCTTAGATTCCGGATTC-3'
IL10	Forward primer	5'-CCCTGGGTGAGAAGCTGAAG-3'
	Reverse primer	5'-CACTGCCTTGCTCTTATTTTCACA-3'
IFIT3	Forward primer	5'-TTCCCAGCAGCACAGAAAC-3'
	Reverse primer	5'-AAATTCCAGGTGAAATGGCA-3'
ISG15	Forward primer	5'-TCCATGACGGTGTCAGAACT-3'
	Reverse primer	5'-GACCCAGACTGGAAAGGGTA-3'
