# Supplementary material for: Quadrivalent mosaic HexaPro-bearing nanoparticle vaccine protects against infection of SARS-CoV-2 variants
Source: Nat Commun. 2022 May 13;13:2674. doi: 10.1038/s41467-022-30222-w (PMC9106700; doi:10.1038/s41467-022-30222-w)
Supplement: Supplementary file 2 — Reporting Summary [file 41467_2022_30222_MOESM2_ESM.pdf]

nature portfolio

Corresponding author(s): Mu-Sheng Zeng

Last updated by author(s): March 24, 2022

Reporting Summary

Nature Portfolio wishes to improve the reproducibility of the work that we publish. This form provides structure for consistency and transparency in reporting. For further information on Nature Portfolio policies, see our Editorial Policies and the Editorial Policy Checklist.

Statistics

For all statistical analyses, confirm that the following items are present in the figure legend, table legend, main text, or Methods section.

n/a	Confirmed	
☐	☒	The exact sample size (*n*) for each experimental group/condition, given as a discrete number and unit of measurement
☐	☒	A statement on whether measurements were taken from distinct samples or whether the same sample was measured repeatedly
☐	☒	The statistical test(s) used AND whether they are one- or two-sided<br>*Only common tests should be described solely by name; describe more complex techniques in the Methods section.*
☒	☐	A description of all covariates tested
☐	☒	A description of any assumptions or corrections, such as tests of normality and adjustment for multiple comparisons
☐	☒	A full description of the statistical parameters including central tendency (e.g. means) or other basic estimates (e.g. regression coefficient) AND variation (e.g. standard deviation) or associated estimates of uncertainty (e.g. confidence intervals)
☐	☒	For null hypothesis testing, the test statistic (e.g. F, t, r) with confidence intervals, effect sizes, degrees of freedom and P value noted<br>*Give P values as exact values whenever suitable.*
☒	☐	For Bayesian analysis, information on the choice of priors and Markov chain Monte Carlo settings
☒	☐	For hierarchical and complex designs, identification of the appropriate level for tests and full reporting of outcomes
☒	☐	Estimates of effect sizes (e.g. Cohen's *d*, Pearson's *r*), indicating how they were calculated

Our web collection on statistics for biologists contains articles on many of the points above.

Software and code

Policy information about availability of computer code

Data collection	GloMax Navigator GM2010 luminometer, EpochTM 2 microplate spectrophotometer, ELISPOT reader
Data analysis	GraphPad Prism version 8.0; Microsoft Excel from Microsoft Office Professional plus 2016, PyMOL-2.5.2, UCSF ChimeraX-1.1.1, Octet Analysis, Uncle Analysis, Studio 12.2.0.20, Sic_axle

For manuscripts utilizing custom algorithms or software that are central to the research but not yet described in published literature, software must be made available to editors and reviewers. We strongly encourage code deposition in a community repository (e.g. GitHub). See the Nature Portfolio guidelines for submitting code & software for further information.

Data

Policy information about availability of data

All manuscripts must include a data availability statement. This statement should provide the following information, where applicable:

- Accession codes, unique identifiers, or web links for publicly available datasets
- A description of any restrictions on data availability
- For clinical datasets or third party data, please ensure that the statement adheres to our policy

All datasets generated or analyzed during this study are included in the figures and supporting information. All other data used in this work are available from the corresponding author on reasonable request. The accession numbers of spike of coronavirus referred in this work are obtained from Genbank or GISAID database as following: Prototypic SARS-CoV-2 HexaPro (GISAID accession ID: EPI_ISL_413859), and England/MILK-9E05B3/2020 (GISAID accession ID: EPI_ISL_601443), South Africa/NHLS-UCT-GS-1067/2020 (GISAID accession ID: EPI_ISL_700428), Japan/IC-0561/2021 (GISAID accession ID: EPI_ISL_792680), hCoV-19/Nigeria/CV844/2021 (GISAID accession ID: EPI_ISL_1235642) and hCoV-19/India/MP-NCDC-2509230/2020 (GISAID accession ID: EPI_ISL_2461258), HCoV-229E (GenBank: APT69883.1), MERS-CoV (GenBank: AFS88936.1), HCoV-NL63 (GenBank: APF29071.1), HCoV-OC43 (GenBank: AVR40344.1.), RATG13 (GenBank: QHR63300.2) and SARS-CoV

(GenBank: AAP13567.1).

Field-specific reporting

Please select the one below that is the best fit for your research. If you are not sure, read the appropriate sections before making your selection.

☒ Life sciences ☐ Behavioural & social sciences ☐ Ecological, evolutionary & environmental sciences

For a reference copy of the document with all sections, see nature.com/documents/nr-reporting-summary-flat.pdf

Life sciences study design

All studies must disclose on these points even when the disclosure is negative.

Sample size	Sample size for immunogenicity assessment studies in mice and cynomolgus macaques were determined by the similar studies from our lab (doi: 10.1021/acsnano.0c08379,doi: 10.1021/acs.nanolett.0c04687, doi: 10.1038/s41421-021-00302-0). Sample size for challenge protection assay in vivo were estimated from the our group performed previously published work (doi: 10.1016/j.cell.2020.06.010). For mice study, the number of tested mice in immunogenicity assessment studies in each group was 5-6. The number of mice in challenge protection assay in vivo in each group was 10. For cynomolgus macaques study, the number of cynomolgus macaques in immunogenicity assessment studies in each group was 4, which is acceptable in the statistical analysis. For ethical reasons, the minimum number of experimental animals necessary to achieve the scientific objectives was used.
Data exclusions	No data were excluded
Replication	Serum ELISA assay, pseudovirus and authentic virus neutralization assay were replicated once. ELISA assay for evaluation the binding of antigen and antibody were performed twice. BLI assay for antigen and antibody/receptor kinetic were performed twice. Cytokine and chemokine mRNAs, SARS-CoV-2 DNA copies in lung were performed in triplicates. Immunoprecipitation assay was performed twice. Replication of experiments were successful.
Randomization	BALB/c mice were randomly divided into 6 groups. Cynomolgus macaques divided into two groups according to body weight. For the challenge protection assay in vivo, the mice for per immunized group (n=20) were randomly divided into two groups (n=10 for each group).
Blinding	No blinding was conducted since there was no specific grouping

Reporting for specific materials, systems and methods

We require information from authors about some types of materials, experimental systems and methods used in many studies. Here, indicate whether each material, system or method listed is relevant to your study. If you are not sure if a list item applies to your research, read the appropriate section before selecting a response.

Materials & experimental systems

n/a	Involved in the study	
☐	☒	Antibodies
☐	☒	Eukaryotic cell lines
☒	☐	Palaeontology and archaeology
☐	☒	Animals and other organisms
☒	☐	Human research participants
☒	☐	Clinical data
☒	☐	Dual use research of concern

Methods

n/a	Involved in the study	
☒	☐	ChIP-seq
☒	☐	Flow cytometry
☒	☐	MRI-based neuroimaging

Antibodies

Antibodies used	REGN-10933, Regdanvimab, S2-E12, COVA1-16, S2-H14, S2-M11, CB6, IgG1-ab1, P2B-2F6, CR3022, COV2-2196, 4A8, Fab 2-15 and REGN-10987 mAbs were derived by gene synthesis (GenScript) from the coronavirus antibody database (http://opig.stats.ox.ac.uk/webapps/covabdab), and produced in our lab using Expi293FTM suspension cells, and then aliquoted and stored as 1 mg/mL in PBS buffer. AMMO1 mAb was derived by gene synthesis (GenScript) from the published sequence in Snijder J, et al. An Antibody Targeting the Fusion Machinery Neutralizes Dual-Tropic Infection and Defines a Site of Vulnerability on Epstein-Barr Virus. Immunity 48, 799-811 e799 (2018).<br>Antibodies used in ELISA assay<br>Horseradish peroxidase (HRP)-conjugated goat anti-human IgG(H+L) secondary antibody (SouthernBiotech, cat. no: 2015-05, 1 : 5000 dilution); HRP-conjugated goat anti-mice IgG(H+L) secondary antibody (Abcam, cat. no: ab6789, 1 : 5000 dilution).<br>Antibodies used in focus forming assay<br>rabbit anti-SARS-CoV-2 nucleocapsid primary antibody (Sino Biological, cat. no: 40143-T62,1 : 12000 dilution)<br>HRP-conjugated goat anti-rabbit secondary antibody (Jackson ImmunoResearch Laboratories, cat. no: 111-035-144, 1 : 6000 dilution).

	Antibodies used in immunohistochemistry<br>rabbit anti-SARS-CoV-nucleoprotein polyclonal antibody (Novus, cat. no: NB100-56576, 1 : 1000 dilution)<br>HRP-conjugated goat anti-rabbit IgG (Abcam, cat. no: ab205718, 1:2000 dilution)
Validation	REGN-10933, Regdanvimab, S2-E12, COVA1-16, S2-H14, S2-M11, CB6, IgG1-ab1, P2B-2F6, CR3022, COV2-2196, 4A8, Fab 2-15 and REGN-10987 mAbs were isolated from convalescent COVID-19 patients, and have been validated previously (Sun et al. Signal Transduction and Targeted Therapy (2022) 7:42; https://doi.org/10.1038/s41392-022-00910-6). AMMO1 mAb was isolated from human, have been validated previously (Snijder J, et al. Immunity 48, 799-811 e799 (2018), https://doi.org/10.1016/j.immuni.2018.03.026). The VH and VK/Vλ genes of the above antibodies were obtained from Genbank and protein data bank database, and then codon optimized genes were synthesized by GenScript, followed by cloned into antibody expression vectors containing the constant regions of human IgG1.<br>HRP-conjugated goat anti-human IgG(H+L) secondary antibody (SouthernBiotech, cat. no: 2015-05, 1 : 5000 dilution) : specific for human IgG, suitable for ELISA and FLISA. (https://www.southernbiotech.com/goat-anti-human-igg-h-l-hrp-2015-05).<br>HRP-conjugated goat anti-mice IgG(H+L) secondary antibody (Abcam, cat. no: ab6789, 1 : 5000 dilution), specific for mice IgG, suitable for ICC, IP, Dot blot, ELISA, IHC-P, IHC-Fr, Immunomicroscopy, WB. (https://www.abcam.com/goat-mouse-igg-hl-hrp-ab6789.html).<br>rabbit anti-SARS-CoV-2 nucleocapsid primary antibody (Sino Biological, cat. no: 40143-T62, 1 : 12000 dilution), suitable for WB and ELISA, and have been validated previously (Sun J, et al. Generation of a Broadly Useful Model for COVID-19 Pathogenesis, Vaccination, and Treatment. Cell 182, 734-743 e735 (2020). https://doi.org/10.1016/j.cell.2020.06.010) (https://www.sinobiological.com/antibodies/cov-nucleocapsid-40143-t62).<br>HRP-conjugated goat anti-rabbit secondary antibody (Jackson ImmunoResearch Laboratories, cat. no: 111-035-144, 1 : 6000 dilution) , suitable for immunoelectrophoresis and/or ELISA, and have been validated previously (Sun J, et al. Generation of a Broadly Useful Model for COVID-19 Pathogenesis, Vaccination, and Treatment. Cell 182, 734-743 e735 (2020). https://doi.org/10.1016/j.cell.2020.06.010. (https://www.jacksonimmuno.com/catalog/products/111-035-144).<br>rabbit anti-SARS-CoV-nucleoprotein polyclonal antibody (Novus, cat. no: NB100-56576, 1 : 1000 dilution) , suitable for WB, ICC/IF, IHC, IHC-P, Dual ISH-IHC, and have been validated previously (Ulrich L, et al. Enhanced fitness of SARS-CoV-2 variant of concern Alpha but not Beta. Nature, (2021), https://doi.org/10.1038/s41586-021-04342-0). (https://www.novusbio.com/products/sars-nucleocapsid-protein-antibody_nb100-56576).<br>HRP-conjugated goat anti-rabbit IgG (Abcam, cat. no: ab205718, 1:2000 dilution), suitable for: IHC-P, WB, ELISA and IP. (https://www.abcam.com/Goat-Rabbit-IgG-HL-HRP-ab205718.html).

Eukaryotic cell lines

Policy information about cell lines

Cell line source(s)	HEK293T (CRL-3216), Vero E6 (CRL-1586), and Vero (CCL-81) were purchased from ATCC. Human ACE2-expressing HEK 293T cells (293T-hACE2) were established in our laboratory which were derived from the HEK293T cell line stably expressing human angiotensin-converting enzyme 2 (hACE2). Expi293FTM cells (A14527) were purchased from Thermo Fisher Scientific.
Authentication	All cell lines used in this study were frequently checked for cellular morphologies, growth rates and functions
Mycoplasma contamination	All cell lines were tested negative for mycoplasma contamination by Polymerase Chain Reaction (PCR)
Commonly misidentified lines<br>(See ICLAC register)	No commonly misidentified cell lines were used.

Animals and other organisms

Policy information about studies involving animals; ARRIVE guidelines recommended for reporting animal research

Laboratory animals	six-week-old specific pathogen-free (SPF) male BALB/c mice were purchased from Zhejiang Vital River Laboratory Animal Technology Co., Ltd, and raised in the laboratory animal facilities at Sun Yat-sen University Cancer Center. Immunized mice (aged 12 weeks old) were exported to the BSL3 facility at the Guangzhou Customs District Technology Center for challenge assays. Mice were housed under standard humidity (55±10%) and temperature (21±1°C) conditions in a 12-hour dark/light cycle with enough food and water.<br>14-month-old cynomolgus macaques (Macaca fascicularis, six females and two males, body weight ranging from 1.5 to 1.8 kilograms) were purchased from Zhanjiang Prima Biotech Inc. and maintenanced at the Primate Research Center of the Institute of Zoology, Guangdong Academy of Sciences.
Wild animals	The study did not involve wild animals.
Field-collected samples	The study did not involve field-collected samples.
Ethics oversight	All of the animal experiments were reviewed and approved by the the Ethics Committee of the Guangzhou Medical University, Sun Yat-sen University Cancer Center and Institute of Zoology, Guangdong Academy of Sciences.

Note that full information on the approval of the study protocol must also be provided in the manuscript.
